# Revealing molecular determinants governing mambalgin-3 pharmacology at acid-sensing ion channel 1 variants

**DOI:** 10.1007/s00018-024-05276-2

**Published:** 2024-06-17

**Authors:** Ben Cristofori-Armstrong, Elena Budusan, Jennifer J. Smith, Steve Reynaud, Kerstin Voll, Irène R. Chassagnon, Thomas Durek, Lachlan D. Rash

**Affiliations:** 1 Australian Institute for Bioengineering and Nanotechnology, The University of Queensland, Brisbane, QLD 4072 Australia; 2 School of Biomedical Sciences, The University of Queensland, Brisbane, QLD 4072 Australia; 3 Institute for Molecular Bioscience, The University of Queensland, Brisbane, QLD 4072 Australia; 4 Department of Biomedical Sciences, University of Lausanne, Lausanne, Switzerland; 5 Genentech, 1 DNA Way, South San Francisco, CA 94080 United States; 6 In Extenso Innovation Growth, Lyon, France; 7 Boehringer Ingelheim Pharma GmbH & Co. KG, Birkendorfer Str. 65, 88397 Biberach an der Riß, Germany; 8 Servatus Ltd. Coolum Beach, Coolum Beach, QLD Australia

**Keywords:** ASIC, Allosteric modulation, Ligand selectivity, Specificity, Venom peptide, Protein-protein interaction, Electrophysiology, Gating modifier

## Abstract

Acid-sensing ion channels (ASICs) are trimeric proton-gated cation channels that play a role in neurotransmission and pain sensation. The snake venom-derived peptides, mambalgins, exhibit potent analgesic effects in rodents by inhibiting central ASIC1a and peripheral ASIC1b. Despite their distinct species- and subtype-dependent pharmacology, previous structure-function studies have focussed on the mambalgin interaction with ASIC1a. Currently, the specific channel residues responsible for this pharmacological profile, and the mambalgin pharmacophore at ASIC1b remain unknown. Here we identify non-conserved residues at the ASIC1 subunit interface that drive differences in the mambalgin pharmacology from rat ASIC1a to ASIC1b, some of which likely do not make peptide binding interactions. Additionally, an amino acid variation below the core binding site explains potency differences between rat and human ASIC1. Two regions within the palm domain, which contribute to subtype-dependent effects for mambalgins, play key roles in ASIC gating, consistent with subtype-specific differences in the peptides mechanism. Lastly, there is a shared primary mambalgin pharmacophore for ASIC1a and ASIC1b activity, with certain peripheral peptide residues showing variant-specific significance for potency. Through our broad mutagenesis studies across various species and subtype variants, we gain a more comprehensive understanding of the pharmacophore and the intricate molecular interactions that underlie ligand specificity. These insights pave the way for the development of more potent and targeted peptide analogues required to advance our understating of human ASIC1 function and its role in disease.

**Supplementary Information:**

The online version contains supplementary material available at 10.1007/s00018-024-05276-2.

## Introduction

Physiological extracellular pH is maintained at pH ~ 7.4, with acidic perturbations sufficient to activate acid-sensing ion channels (ASICs) being strongly associated with the detection and processing of pain [1, 2]. The role of ASICs in pain pathways has been well established by the combined use of genetic models and pharmacological tools [3–5]. Mambalgins are a family of three-finger peptide toxins (Ma-1, Ma-2, and Ma-3) that are reported to have indistinguishable ASIC pharmacology [6, 7]. They were isolated as the first potent dual ASIC1a and ASIC1b inhibitors and were instrumental in revealing that ASIC1b plays a role in peripheral nociception [8]. Recent studies have confirmed that they produce ASIC1b-mediated peripheral analgesia in several animal models of inflammation- and trauma-related pain [9–11].

**Fig. 1:**
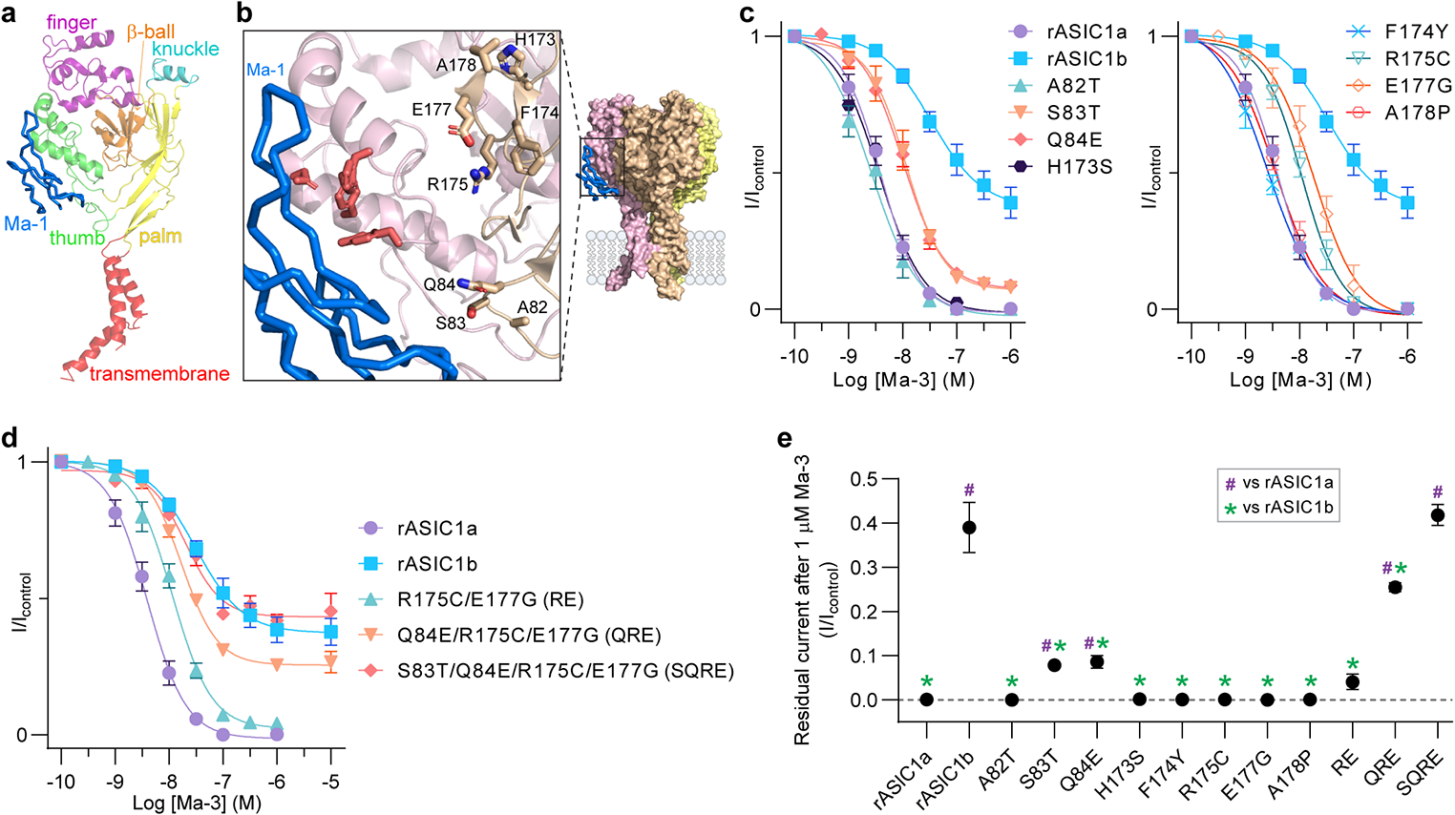
Multiple substitutions combined in rASIC1a alter Ma-3 pharmacology to resemble that at rASIC1b (**a**) Cryo-EM complex of Ma-1 bound to hASIC1a (PDB: 7CFT) showing a single ASIC1a subunit with domains coloured and labelled. (**b**) The complex structure showing: (i) the core pharmacophore of four residues on the thumb domain (red sticks). Although these residues are unlabelled, they are Tyr316, Asn320, Phe350, and Tyr358 using rASIC1a numbering, and (ii) residues at the interface of subunits that were mutated in this study are shown as brown sticks and labelled. Labels show rASIC1a residues which are all the same as hASIC1a except for A178 which is a valine in hASIC1a. (**c**) Concentration-response curves for Ma-3 at individual mutants of rASIC1a, and (**d**) combination mutants of rASIC1a. See Supplementary Table 2 for full Hill equation fits and statistical comparisons. (**e**) Maximal inhibition by 1 µM Ma-3 at each channel tested. All data use a conditioning pH of 7.45 and stimulating pH of 6. Welch’s one-way ANOVA with Dunnett’s multiple comparisons test where *P* < 0.05 is considered significant compared to rASIC1a (purple hash, #) and rASIC1b (green asterisk, *)

Mambalgins are gating modifiers of ASIC1, with pharmacological effects that depend on the subtype and species [8, 12]. They are ~ 5-fold more potent on rat (r) than human (h) ASIC1a, with IC_50_ values for rASIC1a ranging from 3 to 55 nM and for hASIC1a ranging from 18 to 127 nM. This inhibition is achieved via peptide binding to and stabilising the ASIC1a resting state and preventing activation gating, which is seen as a shift in the pH-dependence of activation to more acidic conditions [8, 12]. In contrast, mambalgins only partially and less potently inhibit rASIC1b, with IC_50_ values of 44 to 192 nM [8, 12]. This inhibition has been shown to occur without shifting the pH-dependence of activation, and modestly shifting the pH-dependence of steady-state desensitisation (SSD) to more acidic values [12]. However, a subsequent study showed that mambalgins effect on rASIC1b is more pH-dependent, causing an acid shift of the activation curve and an alkaline shift of the pH-dependence of steady-state desensitisation [7]. Some of these differences in results may be due to different experimental conditions and mambalgin concentration used. Nonetheless, the mechanism of action of mambalgins at rASIC1b is clearly different to ASIC1a. Strikingly, Ma-3 has been shown to potentiate pH 6-stimulated hASIC1b currents while only weakly inhibiting pH 5-evoked currents [12]. At hASIC1b, both the activation and steady-state desensitisation curves are alkaline shifted in the presence of mambalgins [7, 12]. While it is the ASIC1b pharmacology that is relevant to the use of the mambalgins as tools to study the role of ASIC1 in peripheral pain, most studies on the structure-function relationship have focused on the interaction of mambalgins with ASIC1a. Residues Gln5, His6, Lys8, Asn22, Phe27, Arg28, Leu32, Ile33, and Leu34 of Ma-1 comprise the core pharmacophore for rASIC1a [13, 14]. The cryo-EM complex structure of Ma-1 bound to hASIC1a at 3.9Å overall resolution shows the peptide binds predominantly to the thumb domain [15], consistent with functional studies demonstrating that a cluster of four residues (Tyr316, Asn320, Phe350, and Tyr358; rASIC1a numbering) on the thumb domain are crucial for mambalgin activity (see Fig. 1a and b) [14, 16]. These residues are conserved across rat and human ASIC1 subtypes, thus have not revealed which ASIC residues determine the selectivity of mambalgins or whether the mambalgin pharmacophore is the same for ASIC1b and ASIC1a. Understanding the molecular basis of the subtype and species selectivity of the mambalgins is essential for their development and use to understand the roles of ASICs in peripheral pain in both rodents and humans.

In this study, we identify amino acids on both the peptide and channel that determine the different pharmacology of Ma-3 across ASIC1 species and subtype variants. Our findings reveal that the differences in Ma-3 potency and efficacy between rat ASIC1a and ASIC1b arise largely from substitutions of residues at the thumb-palm domain subunit interface that are distant from the primary binding site. The rat to human ASIC1a difference in potency is due to a single point mutation in the lower thumb that is involved in a direct binding interaction with Ma-3. Furthermore, we show that the unique pharmacological profile of Ma-3 at human ASIC1b can be explained by a combination of channel differences involved in direct binding interactions and differences involved in channel gating distant from core peptide: channel interactions (i.e. allosteric substitutions). We also present a panel of Ma-3 alanine mutants and demonstrate that Lys8 and Met16 of Ma-3 exhibit subtype-dependent functional importance. These results have yielded a highly selective ASIC1a inhibitor and provide insights into the molecular determinants of Ma-3 selectivity across ASIC1 subtypes, which could help guide the future development of mambalgin analogues with modified potency and selectivity profiles.

## Materials and methods

### Peptide production

Recombinant Ma-3 and mutants were produced as previously described, using a periplasmic *E. coli* expression system [12, 17]. A synthetic gene encoding Ma-3 was purchased from Invitrogen (Thermo Fisher Scientific, Brisbane, Australia), and mutations were introduced via PCR-based site-directed mutagenesis for variants. All clones were confirmed via Sanger sequencing (Australian Genome Research Facility, Brisbane, Australia). The gene encoding each peptide was cloned into a pLic-polyhistidine (His6)-maltose binding protein (MBP)-tobacco etch virus (TEV) expression vector. This plasmid was transformed into *E. coli* BL21(λDE3) cells, and large-scale growth performed in Terrific Broth medium at 35 °C. Peptide expression was induced by 0.7 mM isopropyl β-D-1-thiogalactopyranoside (IPTG) at OD_600_ = 1.0–1.3, and the cell pellet collected after overnight growth at 18 °C via centrifugation for 10 min at 5000 x *g*. The cell pellet was resuspended in buffer (containing 50 mM Tris, 500 mM NaCl, 5% glycerol, pH 8.3) at 10 mL per gram, then processed via cell disruption at 28 kPSI and 32 kPSI (TS Series Cell disruptor, Constant System Ltd, UK). After centrifugation at 42,000 x *g* for 45 min, the supernatant containing the His6-MBP fusion proteins were purified by passing over Ni-nitrilotriacetic acid (Ni-NTA) resin beads (Thermo Fischer Scientific). Non-specific protein binders were washed off with 15 mM imidazole before eluting the fusion proteins from the beads with 300 mM imidazole. The buffer was exchanged to remove imidazole and the fusion proteins concentrated by centrifugation in a centrifugal filter device (Millipore Amicon Ultra-15 30 K concentrator; Sigma). Concentrated fusion proteins were cleaved by TEV protease (500 µl of 1 mg/ml TEV protease per litre of bacterial culture) overnight to liberate recombinant peptides. To maintain TEV protease activity, 5 mM reduced (GSH) and 0.5 mM oxidised glutathione (GSSG) were added to the reaction. The TEV reaction was acidified by adding trifluoroacetic acid to 1% final volume, the sample equilibrated into high-performance liquid chromatography (HPLC) solvents, then centrifuged and filtered before HPLC purification. Recombinant peptides contain a non-native N-terminal serine that is a vestige of the protease cleavage site, and this residue is not included in the amino acid numbering used here. All peptides are > 95% purity as assessed using HPLC analyses (Supplementary Fig. 1).

### Oocyte electrophysiology

Two-electrode voltage-clamp (TEVC) experiments using *Xenopus laevis* oocytes were conducted as previously described [12, 18]. Briefly, oocytes were surgically removed from anaesthetised female frogs, then treated with collagenase (~ 1 mg/ml, Sigma type I) for 2 h under agitation at room temperature. cRNAs were synthesised using a mMessage mMachine T7 or SP6 transcription kit (Thermo Fisher Scientific Australia Pty Ltd, Scoresby, VIC, Australia) and injected into healthy stage V–VI oocytes at 0.2–20 ng per cell. Different amounts of RNA were used for different wild-type and mutant constructs, as well as to titrate expression levels to record over multiple days and maintaining consistent expression levels. Injected oocytes were incubated at 17 °C for 1–7 days in 50% Leibovitz’s L-15 medium (Gibco, Thermo Fisher Scientific Australia Pty Ltd, Scoresby, VIC, Australia), supplemented with 25 µg/mL gentamicin, 25 µg/mL streptomycin, and 2.5% fetal horse serum. Membrane currents were recorded 2–7 days after cRNA-injection under voltage-clamp at -60 mV (Axon Axoclamp 900 A, Molecular Devices, CA, USA) using two standard glass microelectrodes of 0.5–1 MΩ resistance when filled with 3 M KCl solution. Data were sampled at 2 kHz and filtered at 0.1 kHz using Clampex 10 software (Molecular Devices, CA, USA). A microperfusion system was used to allow fast extracellular solution exchange (bath volume = ~ 30 µL). All experiments were performed at room temperature (18–23 °C) in ND96 (96 mM NaCl, 2 mM KCl, 1.8 mM CaCl_2_, 2 mM MgCl_2_, 5 mM HEPES, pH 7.45). In solutions below pH 6.8, HEPES was replaced by MES. All solutions containing peptides contained 0.05% fatty acid free bovine serum albumin to decrease adsorptive losses of peptide to plasticware. For all channels tested, control current magnitudes between 500 and 9000 nA when tested using a conditioning pH of 7.45 and stimulating pH of 6 (except for the chimeras that were stimulated with pH 5) were used for data collection. Before recording any experiments, oocytes were stimulated multiple times with low pH to minimise and stabilise tachyphylaxis/desensitisation, which were then accounted for during analysis. Complete recovery of currents from Ma-3 induced modulation within 3 min of washout at all channels tested allowed for correction for tachyphylaxis/desensitisation during analyses. All channel mutants tested showed current profiles typical for ASICs.

**Fig. 2:**
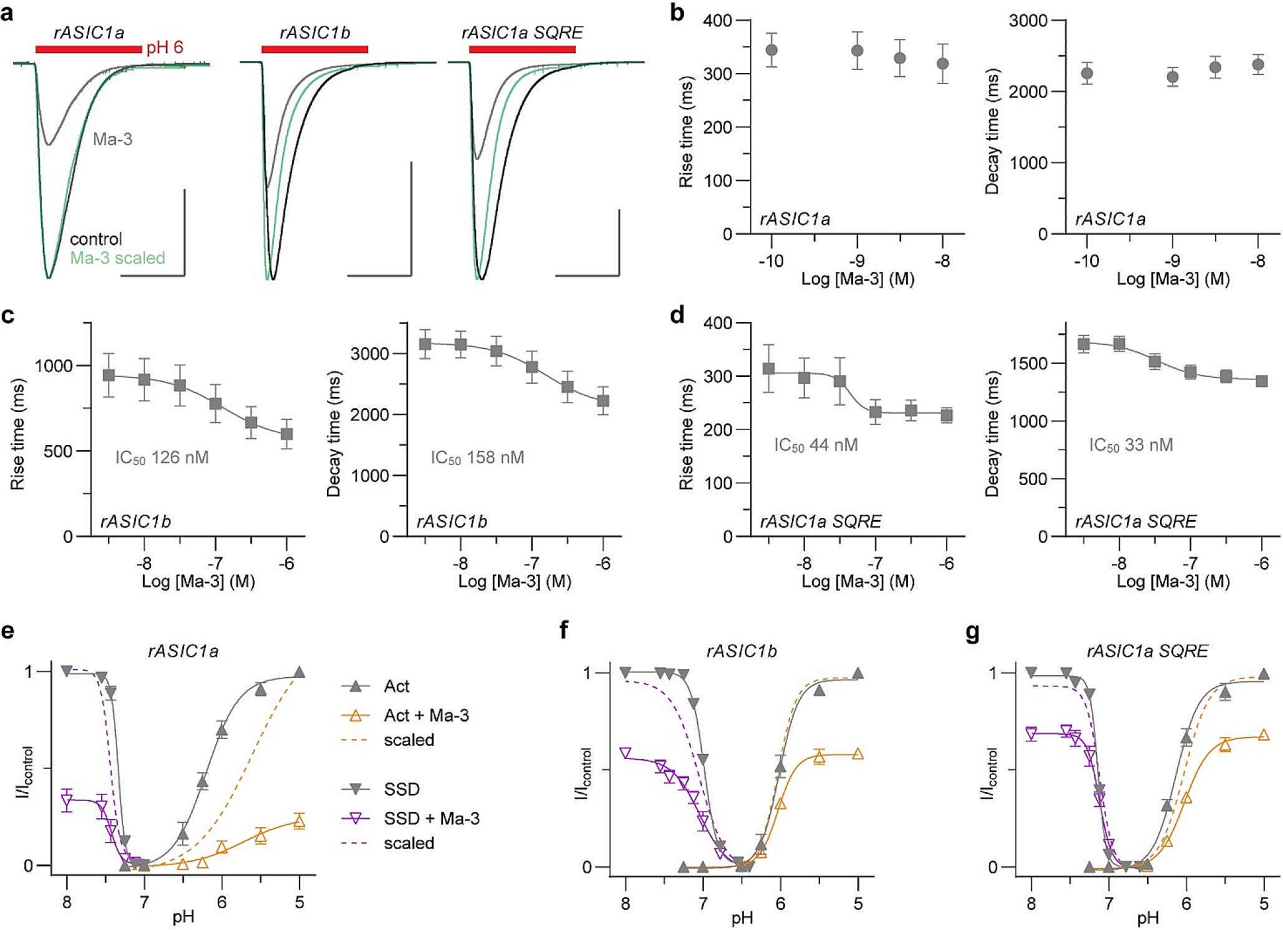
Ma-3 induces distinct changes in the gating properties of rASIC1a, rASIC1b, and the rASIC1a SQRE mutant (**a**) Example traces showing current kinetics after Ma-3 application; 10 nM at rASIC1a, 300 nM at rASIC1b and rASIC1a SQRE mutant. Scale bar: abscissa 3 s, ordinate 1000 nA. (b–d) Average raw rise and decay times analysed from concentration-response data for (**b**) rASIC1a, (**c**) rASIC1b, and (**d**) rASIC1a SQRE mutant (conditioning pH 7.45 and stimulating pH 6). (e − g) The pH dependence of activation (up-pointing triangle, conditioning pH 8) and steady-state desensitisation (SSD; down-pointing triangle, stimulating pH 5) in the absence and presence of 100 nM Ma-3 for (**e**) rASIC1a, (**f**) rASIC1b, and (**g**) rASIC1a SQRE mutant. Details of the Hill equation fits are reported in Table 1. Solid lines represent pH dependence curves normalised to the maximal control current (left y-axis of I/I_control_), and dashed lines are for data scaled to the maximal current observed in the presence of Ma-3. All data are mean ± SEM and *n* = 5–8

**Fig. 3:**
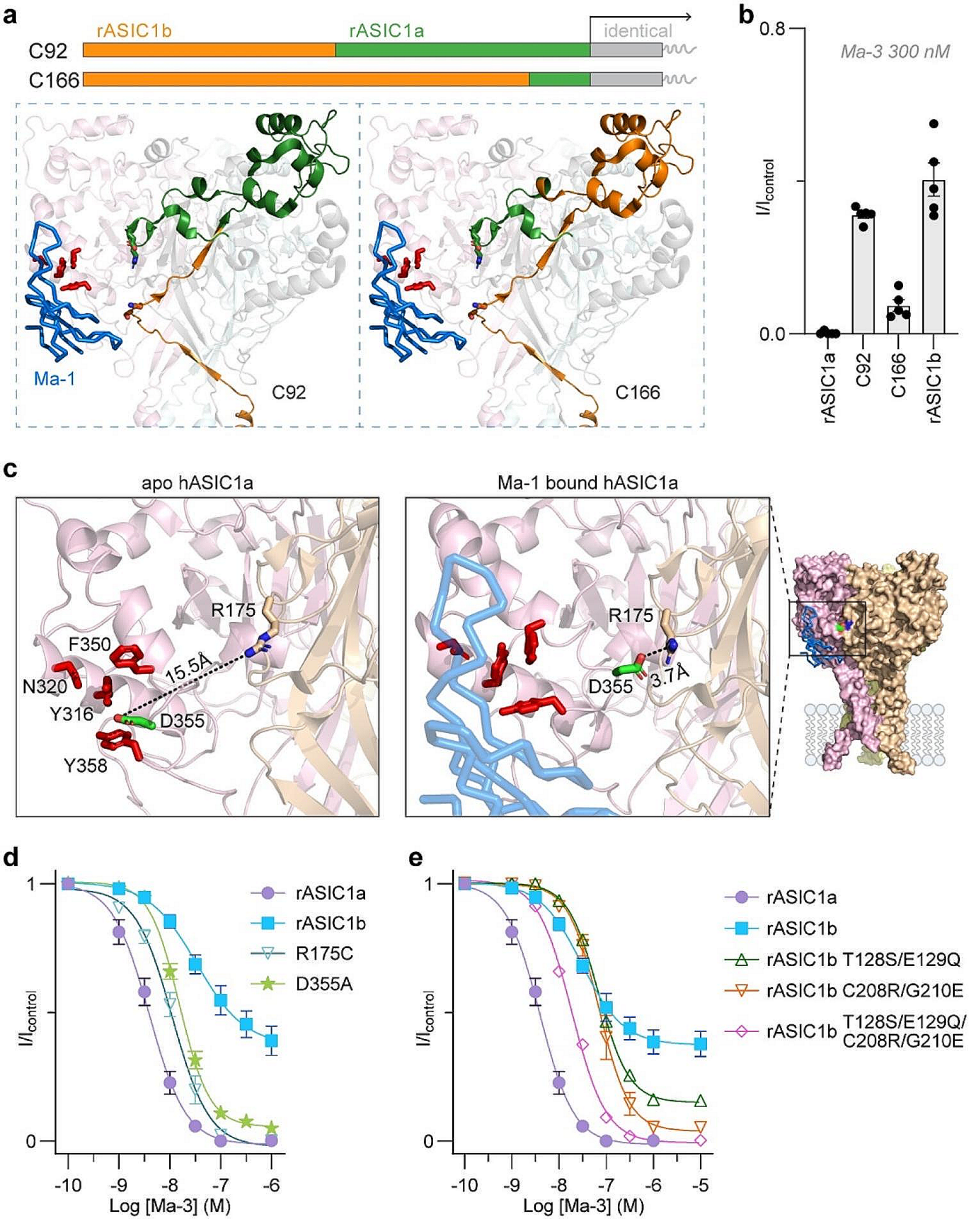
Inter-domain coordination of conformational rearrangements during gating regulates the activity of Ma-3. (**a**) Schematic and structural representation of rat ASIC1a: ASIC1b chimeras. ASIC1a and ASIC1b are identical in the C-terminal two-thirds of the protein (grey box and line in the schematic) and only differ in the first third of the protein (residues 1–186 of rASIC1a and interchanged with rASIC1b). The schematic shows the N-terminal sequences from ASIC1b in orange, and the ASIC1a sequence in green. ASIC1a has a shorter N-terminus than ASIC1b, and the chimeras use the first amino acid of ASIC1a as the starting point. The extended N-terminal sequence of rASIC1b is intracellular. Bottom panels have the chimeric regions mapped onto the Ma-1:hASIC1a complex structure (PDB: 7CFT) of one monomer in the trimer, with the identical region shown in grey (the additional two monomers are coloured pink and cyan). (**b**) Activity of 300 nM Ma-3 at each wild-type and chimeric channel using a conditioning pH of 7.45 and stimulating pH of 5. (**c**) Comparison of the apo hASIC1a structure (PDB: 7CFS) and Ma-1 bound hASIC1a (PDB: 7CFT) highlighting the different position of Asp355 (green stick), and its proximity to Arg175 (brown stick), under these conditions. The structures were aligned, and distance measurements are made in PyMol 2.6. **(d**) Concentration-response curves for Ma-3 at rASIC1a D355A (IC_50_ = 15.38 nM; pIC_50_ 95% CI = 7.76–7.86; slope 95% CI = 1.69–1.26), and comparison to the R175C mutant and wild-type channels. (**e**) Concentration-response curves for Ma-3 at rASIC1b mutants. See Supplementary Table 3 for full Hill equation fits and statistical comparisons. For panels d and e, the conditioning pH was 7.45 and stimulating pH 6. All data in graphs are mean ± SEM and *n* = 5–7

Specific to this work, the standard protocol was for oocytes to be conditioned at pH 7.45 and channels stimulated every 60 s using a 5 s pH 5 or 6 application, unless stated otherwise. For all concentration-response data, peptides were applied for ~ 50 s between each low pH stimulus application. Activation curves were determined by conditioning oocytes at pH 8 (Fig. 2e − g; and Fig. 5a and b) or 7.45 (Fig. 6; and Supplementary Fig. 2d and c) for 55 s and applying low pH stimuli from 7.25 to 5.0 for 5 s. Steady-state desensitisation (SSD) was determined by applying conditioning solutions of pH values from 8 to 6 for 115 s prior to stimulation by a pH drop to 5 for 5 s. Ma-3 was only applied in the conditioning solution for activation and steady-state desensitisation curves in the presence of peptide. Control pH-dependence curves were determined in the absence of any Ma-3. When assessing the effect of Ma-3 on the rASIC1a: ASIC1b chimeras (C92 and C166; and for the comparative wild-type data), the conditioning pH was 7.45 and stimulating pH 5 (Fig. 3b). Specific details of the conditioning and stimulating pH are noted in each figure legend.

**Fig. 4:**
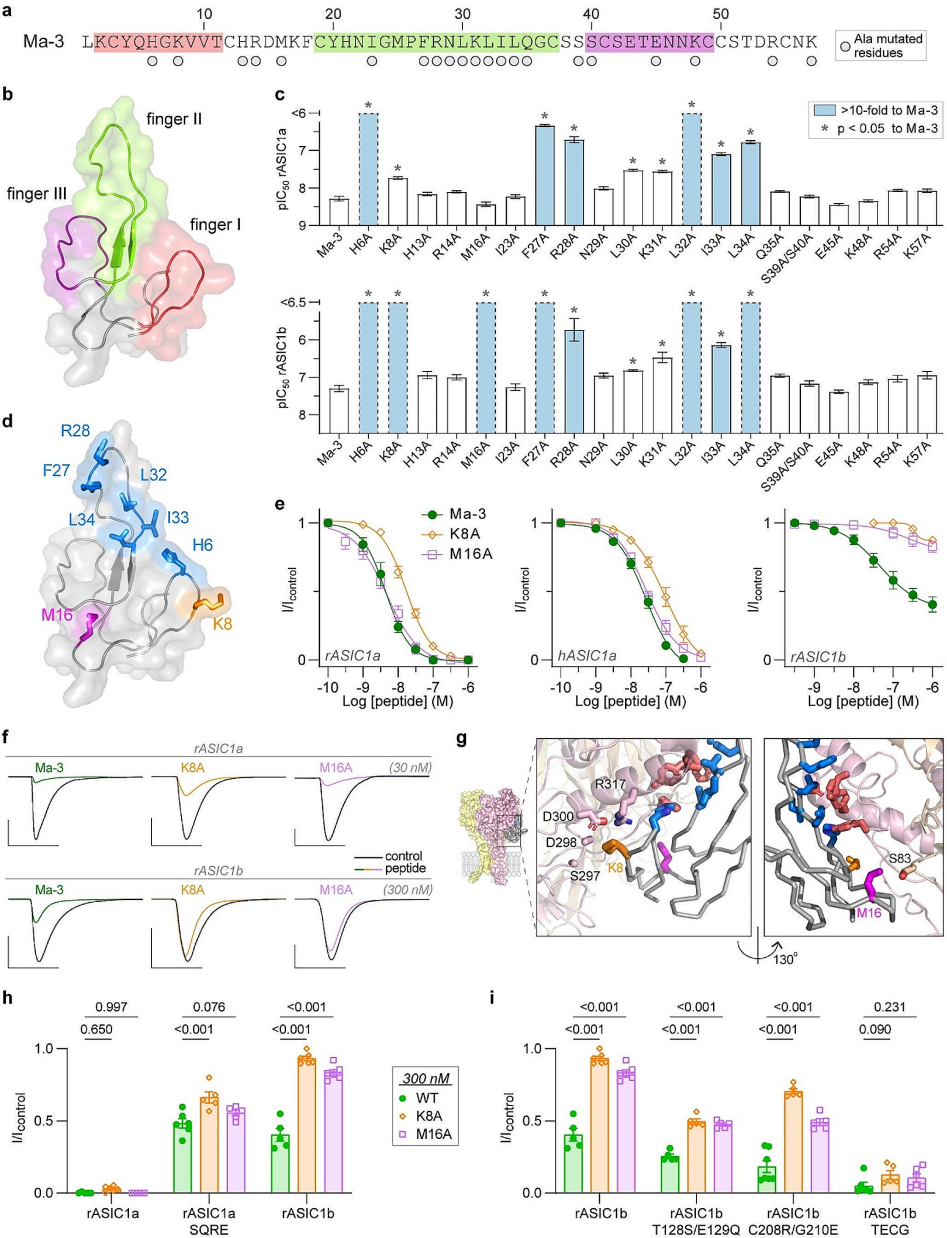
Comparison of the Ma-3 pharmacophore between rASIC1a and rASIC1b. (**a**) Amino acid sequence of Ma-3, with circles below residues that were mutated to alanine in this study. The background colouring highlights the residues that form the three finger loops of mambalgins and are colour coordinated with panel b. (**b**) Structure of Ma-1 from the cryo-EM complex model (PDB: 7CFT) highlighting the three finger loops. (**c**) pIC_50_ values from Hill equation fits for concentration-response data of Ma-3 and mutants at rASIC1a (upper panel) and rASIC1b (lower panel). Dashed bars represent data for which complete fits are not possible due to lack of higher concentration data (see Supplementary Fig. 4). Statistics with Welch’s one-way ANOVA with Dunnett’s multiple comparisons test. (**d**) Ma-1 structure as in panel b, with side chains of residues that show a > 10-fold loss in IC_50_ values compared to wild-type for rASIC1a and rASIC1b are shown in blue, Lys8 in orange, and Met16 in purple. (**e**) Concentration-response curves for Ma-3 wild type, K8A, and M16A at rASIC1a, hASIC1a and rASIC1b. (**f**) Example current traces at rASIC1a (30 nM peptide) and rASIC1b (300 nM peptide). Scale bar: abscissa 4 s, ordinate 500 nA. (**g**) Complex structure highlighting labelled channel residues within 5 Å of K8 (orange, left box) and M16 (purple, right box). (**h**) Activity of 300 nM Ma-3 WT (wild-type), K8A, and M16A mutants at rASIC1a, rASIC1a SQRE (S83T/Q84E/R175C/E177G), and rASIC1b. (**i**) Activity of peptides as in panel h, towards rASIC1b mutants (note: TECG is T128S/E129Q/C208R/G210E and the reverse mutant to rASIC1a SQRE). *P* values above bars in panels h and i, are determined by a two-way ANOVA with Dunnett’s correction for multiple comparisons. All data use a conditioning pH of 7.45 and stimulating pH of 6, are *n* = 5–8, and with full Hill equation fits and statistical comparisons reported in Supplementary Tables 4–6

**Fig. 5:**
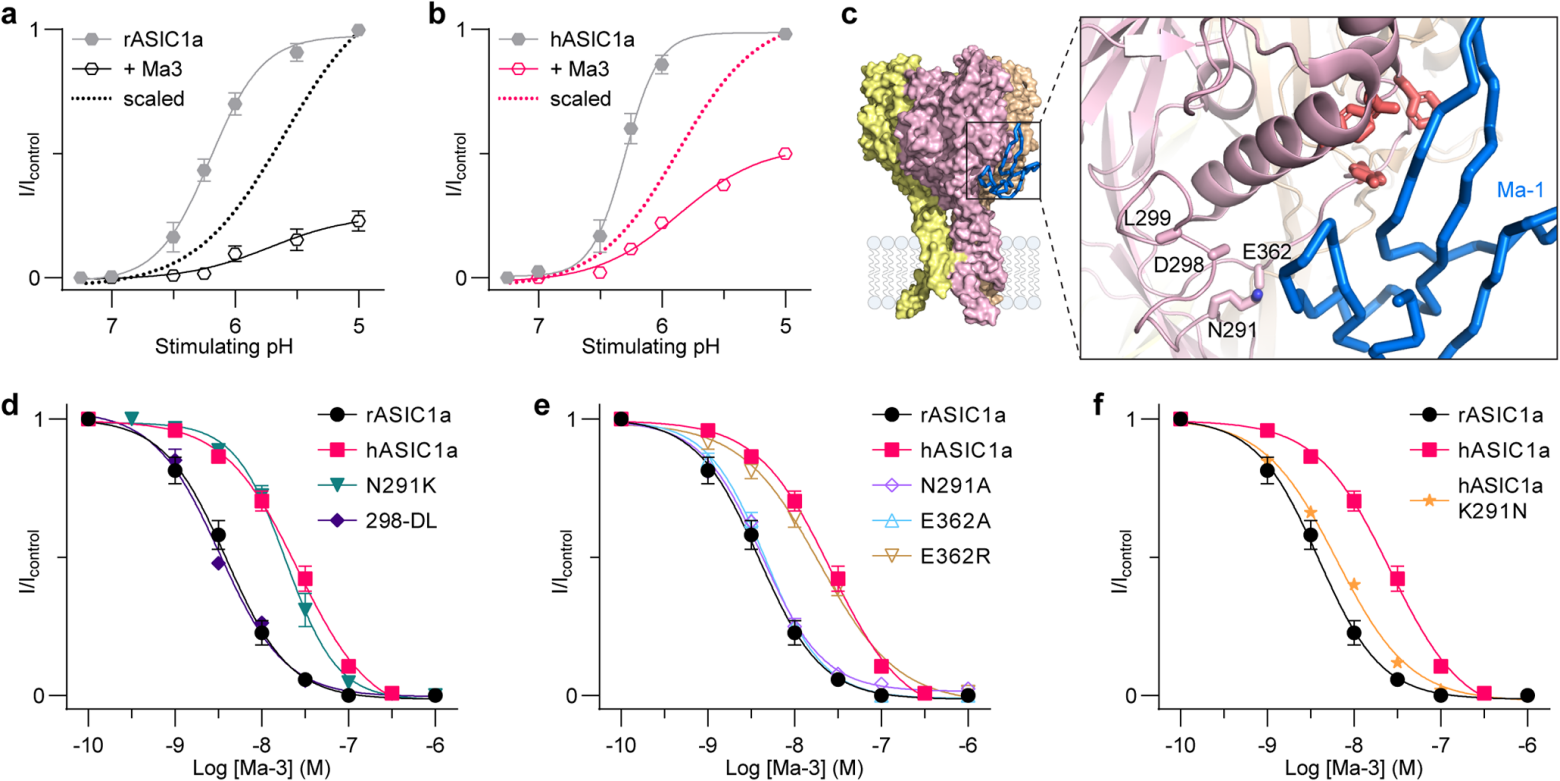
Mutation of residue 291 in the lower thumb domain can explain the Ma-3 potency shift between rat and human ASIC1a. (a–b) The pH-dependence of activation for (**a**) rASIC1a and (**b**) hASIC1a in control (grey), in the presence of 100 nM Ma-3 in the pH 8 conditioning solution (black and pink), and 100 nM Ma-3 scaled to its own maximum (dotted line without symbols). (**c**) Cryo-EM complex of Ma-1 bound to hASIC1a (PDB: 7CFT) with mutated residues labelled using rASIC1a numbering. Asn291 of rASIC1a is a Lys in hASIC1a, and the Asp298 and Leu299 residues shown are insertions in hASIC1a relative to rASIC1a (side chains not built in the cryo-EM model). (e–f) Concentration-response curves for Ma-3 at (d) rASIC1a individual substitutions that differ between species variants, (**e**) local mutations in rASIC1a spatially close to position 291, and (**f**) the reverse substitution at position 291 in the hASIC1a background (all concentration-response data use a conditioning pH of 7.45 and stimulating pH of 6). All data are mean ± SEM and *n* = 5–6. See Supplementary Tables 7–9 for full Hill equation fits and statistical comparisons

### Data and statistical analysis

Data were analysed using pCLAMP-Clampfit software (Version 11, Molecular Devices, CA, USA) and GraphPad Prism 9.5 and 10.2.2 (GraphPad Software, San Diego, CA, USA). To obtain the Hill coefficient (n_H_) and half-maximal response (EC_50_, IC_50_, or pH_50_) the Hill equation was fitted to normalised concentration-response curves by using a non-linear fit to the data of a four-parameter logistic equation (“sigmoidal dose-response” in GraphPad Prism 9.5). The rate of onset of Ma-3 inhibition, and the rate of recovery after Ma-3 inhibition with peptide washout, were fit with an exponential one-phase association or decay equation to obtain t_1/2_ values. Rise and decay times were analysed as 10 to 90% of the full current amplitude (Clampfit 11). For statistical testing, *P* values were calculated via a Welch’s unpaired t-test (comparison of two conditions) or one-way ANOVA (followed by Dunnett’s multiple comparisons for > 2 conditions). Statistical testing of grouped data sets (i.e. Fig. 4h and i), are determined by a two-way ANOVA with Dunnett’s correction for multiple comparisons. Due to limitations with peptide expression yields, higher concentration data were not obtained for several Ma-3 mutants at rASIC1a and rASIC1b. This prevents accurate fitting of the Hill equation and results in pIC_50_ values with considerable error. Given the sigmoidal nature of these data, in order to perform statistical analyses of Ma-3 mutants’ activities the following criteria was applied: (i) if inhibition was < 25% at 1 µM, pIC_50_ was set to 5.5 and (ii) the standard deviation was estimated by taking the average standard deviation of all other data sets being compared that were fit unambiguously with the Hill equation. For all data *P* < 0.05 was considered as the threshold for significance. All data points in graphs are shown as mean ± standard error (SEM). Supplementary tables report pIC_50_ and Hill slope values as 95% CI, and the number of replicates (n) represents separate experimental oocytes.

**Fig. 6:**
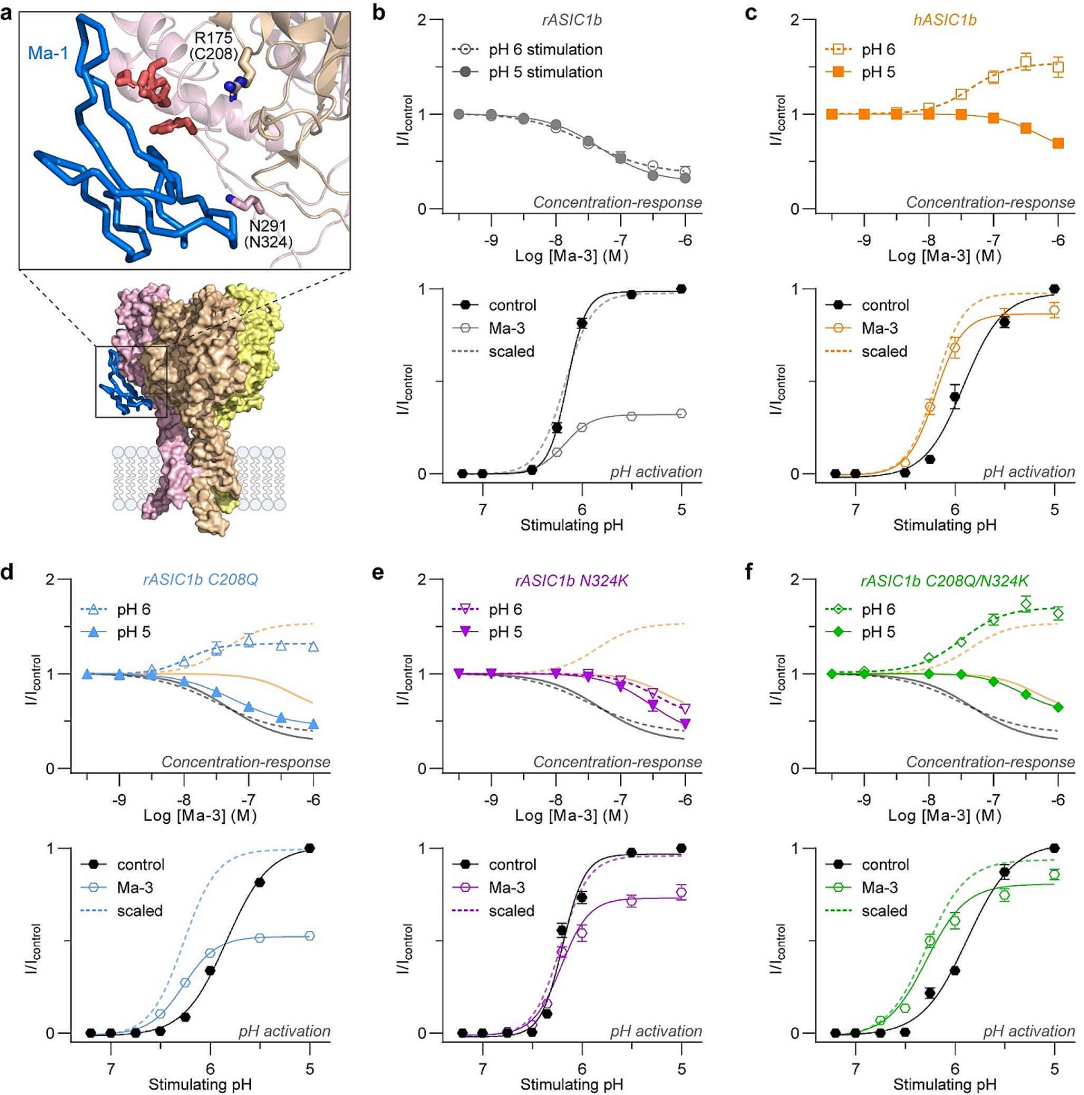
Two amino acid substitutions define the unique pharmacology of Ma-3 at hASIC1b. (**a**) Cryo-EM complex of Ma-1 bound to hASIC1a (PDB: 7CFT). Residues that determine the rat to human ASIC1b selectivity are shown as sticks and labelled. Labels use rASIC1b residues and numbering. Cys208 is a Gln in hASIC1b, and the equivalent of Arg175 in hASIC1a. Asn324 is a Lys in hASIC1b, and the equivalent of Lys291 in hASIC1a. (b–f) Concentration-response curves (top) and pH-dependence of activation (bottom) for (**b**) rASIC1b, (**c**) hASIC1b, (**d**) rASIC1b C208Q, (**e**) rASIC1b N324K, and (**f**) rASIC1b C208Q/N324K. Concentration-response curves are performed using pH 6 stimulus (open symbols and dashed lines) and pH 5 stimulus (solid symbols and lines). pH-dependence of activation data is shown for control (black; each respective channel tested for that figure panel in the absence of Ma-3 – i.e. for panel b, control is rASIC1b without Ma-3), in the presence of 300 nM Ma-3 (coloured), and 300 nM Ma-3 scaled to its own maximum (coloured dashed line without symbols). All data use a conditioning pH of 7.45, are mean ± SEM, and *n* = 5–6. See Supplementary Tables 10 and 11 for full Hill equation fits and statistical comparisons

## Results

### Substitutions at two distant sites along the thumb-palm domain subunit interface are determinants of differences in Ma-3 activity between rat ASIC1a to ASIC1b

We set out to investigate the molecular basis for the different pharmacology of Ma-3 between rat (r) ASIC1a and rASIC1b, which only differ in the first third of the protein (residues 1–186 in rASIC1a) and share an identical thumb domain containing the principal binding site for mambalgins. First, we confirm that the mambalgin variants Ma-1 and Ma-3 (single T23I substitution) show no significant difference in ASIC pharmacology (Supplementary Fig. 2 and Supplementary Table 1). Ma-3 inhibits rASIC1a ~ 10-fold more potently than rASIC1b under the same pH conditions, with IC_50_ values of 3.9 nM and 38.3 nM, respectively (conditioning pH 7.45 and stimulating pH 6; Fig. 1, statistics in Supplementary Table 2). Furthermore, whereas rASIC1a is completely inhibited at saturating Ma-3 concentrations, the inhibition of rASIC1b is incomplete, reaching only ~ 60% inhibition (Fig. 1c and d). To elucidate the molecular basis of this subtype-dependent activity, we targeted non-conserved residues at the interface between the thumb domain and the palm domain of the adjacent subunit, away from the core mambalgin-binding residues on the thumb, changing the residue in rASIC1a to the corresponding residue from rASIC1b (Fig. 1a–e and Supplementary Table 2). Ma-3 showed equipotent inhibition of the single mutations A82T, H173S, F174Y, and A178P compared to wild-type rASIC1a. However, mutants S83T, Q84E, R175C, and E177G were slightly less sensitive to inhibition by Ma-3. These four residues can be spatially grouped with Ser83 and Gln84 located in the lower palm region that is involved in regulating channel desensitisation kinetics [19], and Arg175 and Glu177 in the upper palm region within the acidic pocket. Notably, S83T and Q84E showed incomplete inhibition at maximal concentrations of Ma-3 (~ 90% rather than full inhibition). Nevertheless, no individual mutation could fully explain the subtype-dependent potency and efficacy of Ma-3. Therefore, we sequentially combined mutations until we generated the quadruple mutant (rASIC1a S83T/Q84E/R175C/E177G, or SQRE) (Fig. 1d and e). With the incorporation of each additional mutation, the Ma-3 concentration-response curve progressively resembled that of rASIC1b, with the pharmacological effects of Ma-3 on the quadruple mutant also closely matching its effects on rASIC1b.

We also investigated whether differences in kinetics of Ma-3 activity and the functional state of channels during peptide application could contribute to the observed pharmacological difference of Ma-3 on rASIC1a and rASIC1b. We evaluated the rate of onset of Ma-3 inhibition by applying peptide at ~ 10 times the IC_50_ to channels at pH 7.45 for different durations. We then measured inhibition by activating channels at pH 6. Maximal inhibition was achieved within 45 s of Ma-3 application at both subtypes, and the time course of inhibition could be fit with a single exponential function to give t_1/2_ values of 6.5 s for rASIC1a and 3.8 s for rASIC1b (Supplementary Fig. 3a and b). Notably, complete inhibition of rASIC1b currents was not observed even after 120 s of exposure to Ma-3. We next assessed the recovery of currents from inhibition by Ma-3 at the same concentrations, using pH 7.45 during the conditioning period. This was comparable between the two subtypes with > 75% of current recovered after 60 s of washout of both channels (rASIC1a t_1/2_ = 21.6 s; rASIC1b t_1/2_ = 12.8 s; Supplementary Fig. 3a and b). This result agrees with the idea that variations in the activity profile of Ma-3 between rASIC1a and rASIC1b are not due to differences in the kinetics of inhibition or washout under the conditions used for collecting the data in Fig. 1.

Ma-3 is a gating modifier of ASIC1, and the different pharmacology between rASIC1a and rASIC1b has been tested using the same conditioning and stimulating pH for both channels. However, the pH-dependence of activation and steady-state desensitisation differs between rASIC1a and rASIC1b, with rASIC1b having a lower proton sensitivity by ~ 0.25 pH units (Supplementary Fig. 3c and d). The conformational state of ASICs depends on the pH, and can exist in any of the closed, desensitised, or open states, with the steady-state transitions studied via pH curves. We reevaluated the activity of Ma-3 at a single concentration for inhibition of rASIC1a and rASIC1b at different pH values. This is to compare the equivalent relative conditioning and stimulating pH for each subtype. Changing the conditioning pH from 7.45 to 7.75 did not significantly alter the degree of inhibition observed for rASIC1a (Supplementary Fig. 3e). For rASIC1b, a greater degree of current inhibition was only observed at a conditioning pH where the population of channels being studied started to undergo steady-state desensitisation (Supplementary Fig. 3f). Under the equivalent conditioning pH (pH_50 SSD_ + 0.45 pH units) and activating rat ASIC1a and ASIC1b each with their respective half-maximal activating pH, we still observed a significant difference in the efficacy of inhibition between subtypes (Supplementary Fig. 3g). Therefore, we conclude that the observed pharmacological differences of Ma-3 between rASIC1a and rASIC1b is unlikely to be from the pH conditions used for testing alone. Instead, we propose a more complex mechanism where the predominant binding of Ma-3 to rASIC1a or rASIC1b occurs to a different channel conformation, and once bound the peptide stabilises a different conformation. This is consistent with our findings that residues on the channel in regions known to be important for proton gating underlying the subtype-dependent pharmacology of Ma-3.

To gain further insights into the mechanistic basis for the difference in Ma-3 activity at rASIC1a, rASIC1b, and the rASIC1a SQRE mutant, we analysed the rise and decay times of currents from these channels in the concentration-response data. While the rise and decay times of rASIC1a were not significantly affected by Ma-3, both rASIC1b and the SQRE mutant showed concentration-dependent decreases in rise and decay times (Fig. 2a–d). Where the peptide altered the current kinetics, this effect was reversible and returned to baseline levels with the same timescale as the recovery from inhibition of peak current amplitudes. To further examine differences in how Ma-3 modulates these channels, we determined the pH of activation and steady-state desensitisation (SSD) curves in the absence and presence of 100 nM Ma-3 (Fig. 2e–g; Table 1). At rASIC1a, Ma-3 inhibited the channel by shifting the activation curve by 0.48 pH units towards more acidic values and causing a slight alkaline shift of 0.08 pH units in the SSD curve. In contrast, at rASIC1b, there was no shift in the activation curve, but the SSD curve shifted by 0.06 pH units towards alkaline values. The rASIC1a SQRE mutant exhibited intrinsic pH-gating properties and Ma-3 modulation that were intermediate between the two wild-type channels. Specifically, the control pH_50_ of activation in the SQRE mutant was more similar to that of rASIC1a, while the control pH_50_ of SSD was closer to that of rASIC1b (Table 1). Additionally, 100 nM Ma-3 shifted the activation curve in the SQRE mutant by 0.11 pH units towards acidic values, while the pH_50_ of the SSD curve remained unchanged. While the SQRE mutations appear to bestow the rASIC1b Ma-3 concentration-response phenotype (see Fig. 1), their effects on gating kinetics and pH-properties present a more nuanced profile that combines features of both receptor subtypes. The data on the mechanism of action underscore the allosteric nature of mambalgins and demonstrate how the inherent gating properties of different ASIC variants significantly influence how they are modulated by the peptide.

**Table 1:** Effect of Ma-3 on the pH-dependence of rASIC1a, rASIC1b, and rASIC1a SQRE mutant

	pH_50_	slope
*rASIC1a*		
Act control	6.20 ± 0.03	2.05 ± 0.29
Act + Ma-3	5.72 ± 0.23	1.29 ± 0.67
SSD control	7.34 ± 0.01	9.84 ± 0.67
SSD + Ma-3	7.42 ± 0.04	7.09 ± 1.34
*rASIC1b*		
Act control	6.02 ± 0.02	3.46 ± 0.56
Act + Ma-3	6.03 ± 0.02	3.69 ± 0.56
SSD control	6.98 ± 0.01	5.14 ± 0.24
SSD + Ma-3	7.04 ± 0.04	2.41 ± 0.51
*rASIC1a SQRE*		
Act control	6.14 ± 0.02	2.90 ± 0.33
Act + Ma-3	6.03 ± 0.02	2.68 ± 0.34
SSD control	7.14 ± 0.01	8.54 ± 0.48
SSD + Ma-3	7.12 ± 0.01	5.47 ± 0.95

Fit of the Hill equation to activation (Act) and steady-state desensitisation (SSD) data in the absence (control) and presence of 100 nM Ma-3 (+ Ma-3) to give the pH_50_ and slope. Data are mean ± SEM.

We further investigated the mechanism of ASIC modulation by Ma-3 using two rASIC1a: rASIC1b chimeras, in which increasing segments of rASIC1b were introduced to replace corresponding rASIC1a sequences (C92 and C166, where the number represents the amino acids replaced from the rASIC1a N-terminus; Fig. 3a and b) [20, 21]. Beyond residue 186, both rASIC1a and rASIC1b share the same sequence. The C92 chimera includes the S83T and Q84R substitutions, and 300 nM Ma-3 only partially inhibited this channel, similar to its activity at rASIC1b. Unexpectedly, Ma-3 inhibited a greater proportion of current from the C166 chimera, resembling the activity observed with rASIC1a. Although C166 retains only 19 amino acids unique to rASIC1a, this region includes the palm loop with residues Arg175 and Glu177 from rASIC1a. This data could be interpreted to suggest that the sequence between positions 92 and 166 contains residues that are important in determining mambalgin interactions with the channel. However, we hypothesise that altering this region may change how proton-induced conformational changes during gating are conveyed through the channel, and mambalgins alter these properties in an unpredictable manner. Similar unexpected findings were observed for Ma-2 modulation of ASIC1a: ASIC2a chimeras that exchanged parts of the thumb and palm domains [22]. The unexpected results from the chimera data, together with Ma-3 primarily affecting ASIC1a activation gating, prompted us to compare the thumb-palm domain interface in the apo and Ma-1 bound hASIC1a cryo-EM structures (Fig. 3c). In the apo hASIC1a structure, Asp355 on the thumb domain is within 6Å of the four core ASIC pharmacophore residues, and over 15Å away from Arg175 (distance between side chains). In contrast, in the Ma-1 bound complex, Asp355 shifts to within 4Å of Arg175, possibly forming electrostatic and hydrogen bond interactions that stabilise a thumb: palm domain interaction and prevent further conformational rearrangements associated with activation gating. Although Asp355 does not directly interact with Ma-1 in the bound structure, the Ma-3 concentration-response curve for rASIC1a D355A reveals reduced potency compared to rASIC1a and decreased efficacy at higher concentrations (Fig. 3d). This is consistent with findings from subtype substitutions and chimeras, where domain interface mutations disrupt mambalgin’s ability to inhibit conformational rearrangements during activation gating. Lastly, focusing on the four residues that were crucial for imparting differential Ma-3 potency between rASIC1a and rASIC1b, we generated reverse mutations in the rASIC1b background (Fig. 3e). The rASIC1b T128S/E129Q (reversal of rASIC1a S83T/Q84E) and rASIC1b C208R/G210E (reversal of rASIC1a R175C/E177G) mutants had IC_50_ values similar to rASIC1b but exhibited more current inhibition at saturating concentrations. We then combined the four substitutions to create the reversal of the rASIC1a SQRE mutant. The rASIC1b quadruple mutant showed an IC_50_ of 18.5 nM, intermediate between rASIC1a and rASIC1b, with Ma-3 completely inhibiting currents at saturating concentrations, similar to its effect at rASIC1a.

### Differences in the Ma-3 pharmacophore between rat ASIC1a and rASIC1b

Having confirmed that Ma-3 has a different mechanism of action on rASIC1a and rASIC1b and identifying the channel residues underlying this effect, we next investigated if there was any difference in the Ma-3 pharmacophore for rat ASIC1a and ASIC1b by testing a panel of 20 Ma-3 variants at both subtypes (Fig. 4a). Mambalgins are members of the three-finger toxin family, consisting of three finger loops protruding from a central core (Fig. 4a an b). To test our Ma-3 variants, peptides were applied at pH 7.45 and channels were stimulated at pH 6 (Fig. 4, Supplementary Figs. 1 and 4 and Supplementary Tables 4 and 5). Consistent with previously published reports on activity at rASIC1a [13, 14], we observed an increase in IC_50_ values of greater than 10-fold for Ma-3 H6A in finger I, as well as F27A, R28A, L32A, I33A, and L34A in finger II. Residues in finger III (Ser39, Ser40, Lys48 and Arg54) that are spatially distant from the core pharmacophore of Ma-3 are not important for activity at rASIC1a. Next, we evaluated the activity of our Ma-3 mutants at rASIC1b, where the pharmacophore has not been previously analysed. All alanine mutants that lost activity at rASIC1a also lost activity at rASIC1b, indicating a largely conserved pharmacophore between the two subtypes. However, we observed a significant increase in pIC_50_ at rASIC1b (estimated at > 30-fold loss in potency) for K8A and M16A, in contrast to the modest effect these mutations had on the potency of Ma-3 at rASIC1a and hASIC1a (~ 4-fold for K8A, and no change for M16A) (Fig. 4d–f). Notably, these two residues are located outside the core Ma-3 pharmacophore that is shared between rASIC1a and rASIC1b and are spatially very distant from each other (Fig. 4d). This also positions Lys8 and Met16 relatively distant from channel residues that have been identified as the core binding interactions for potency at ASIC1a (Fig. 4g) [15, 16, 22].

At 300 nM, WT, K8A and M16A Ma-3 strongly inhibit rASIC1a currents. All three peptides showed similar levels of current inhibition (although significantly different for K8A; I/I_control_: WT = 0.48, K8A = 0.65, M16A = 0.56), as wild-type Ma-3 at the rASIC1a SQRE mutant, in contrast to largely abolishing Ma-3’s inhibitory activity as they do at rASIC1b (I/I_control_: WT = 0.40, K8A = 0.91, M16A = 0.80) (Fig. 4h). We then tested these peptides for activity at three rASIC1b mutants (Fig. 4i). At rASIC1b T128S/E129Q, and rASIC1b C208R/G210E, the K8A and M16A mutants were significantly less potent than wild-type Ma-3, akin to the results seen when testing the peptide mutants against wild-type rASIC1b. However, when assaying the peptides at the rASIC1b combined quadruple mutant (TECG), there was minimal difference in inhibition levels between Ma-3 WT, K8A, and M16A. Therefore, the TECG mutant behaved more like rASIC1a, where alanine mutations of Ma-3 at positions Lys8 and Met16 have little effect on peptide potency. Interestingly, the rASIC1a SQRE mutation could effectively swap the lower potency and efficacy of WT Ma-3 at rASIC1b into rASIC1a, with full recovery of efficacy but only partial recovery of potency with the reciprocal mutations in rASIC1b TECG. In contrast, these quadruple channel mutations could only swap the striking subtype dependent effects of Ma-3 K8A and M16A peptide mutations when introduced into the rASIC1b background (TECG mutant), but not reciprocally when the rASIC1b residues were introduced into rASIC1a (SQRE mutant). Figure 2 shows that Ma-3 has very different effects on rASIC1a gating compared to rASIC1b. The lack of correlation between findings in the reciprocal quadruple mutants with Ma-3 WT, K8A, and M16A further supports the idea that mambalgins bind to these two subtype variants in a subtly different orientation and stabilise distinct non-conducting states to achieve inhibition.

### Binding interactions in the lower thumb domain determine the rat to human ASIC1a potency difference for Ma-3

Ma-3 inhibits both rat and human ASIC1a via shifting the pH-dependence of activation curve to more acidic values (Fig. 5a and b; Supplementary Table 7). Furthermore, these species variants are 98% identical in sequence [8, 12, 15], yet rat and human ASIC1a exhibit a difference in IC_50_ values of approximately 6-fold in our experiments (rASIC1a IC_50_ 3.9 nM and hASIC1a IC_50_ 25.0 nM, statistics in Supplementary Table 8). This is not due to differences in the time-course of Ma-3 induced inhibition, or recovery of current from inhibition, which are both comparable between hASIC1a and rASIC1a (Supplementary Fig. 5). Comparison of the amino acid sequences around the Ma-3 binding site reveals two areas that may contribute to the difference in potency (Fig. 5c). To investigate this, we introduced mutations in rASIC1a corresponding to the residues in human ASIC1a at these positions: N291K, D298, and L299 (Fig. 5d). Human ASIC1a contains an Asp298 and Leu299 insertion relative to rASIC1a. Inserting this DL sequence into the rASIC1a background (mutant named 298-DL) had no effect on Ma-3 potency compared to wild-type rASIC1a. However, we found that with the introduction of a positive charge in rASIC1a N291K, the potency of Ma-3 shifted to overlap with that of hASIC1a. In contrast, the alanine mutant at this position, rASIC1a N291A, had no effect on potency (Fig. 5e). To further validate the importance of this channel region, we mutated the spatially neighbouring Glu362 to alanine or arginine to investigate the role of charge at this position (Fig. 5c and e). Ma-3 was equipotent on wild-type and rASIC1a E362A but introducing a positive charge in rASIC1a E362R resulted in a ~ 5-fold decrease in Ma-3 activity. Lastly, we produced the reverse position 291 mutation in the hASIC1a background (hASIC1a K291N), which was inhibited by Ma-3 with a potency comparable to that at wild-type rASIC1a (Fig. 5f). These results suggest that the presence of either a positive charge or additional bulk in the region surrounding Asn291 and Glu362 disrupts Ma-3 activity at ASIC1a through a repulsive and/or steric clash effect. This observation shows that the lower thumb domain contributes to the interaction between ASICs and mambalgins, and the substitution between species variants at position 291 determines rat to human ASIC1a potency of Ma-3. With publication of the Ma-1:hASIC1a cryo-EM structure, it was suggested Lys8 from mambalgin can form a salt bridge interaction with Asp300 of hASIC1a [15], however we find this explanation to be unlikely from our results detailed above.

### Combined binding and allosteric substitutions determine the rat to human ASIC1b difference in Ma-3 activity

The activity and mechanism of action of mambalgins at hASIC1b is different to rat and human ASIC1a and rASIC1b. First, we confirmed that Ma-3 inhibits pH 5 and 6 evoked rASIC1b currents equally and we observed no significant shift in the activation curve with 300 nM peptide application (Fig. 6a–c). In contrast, at hASIC1b, Ma-3 inhibits pH 5 evoked currents and potentiates pH 6 evoked currents, an effect underpinned by an alkaline shift in the pH-dependence of activation (Fig. 6c). To explore the molecular basis for this distinct pharmacology, we examined the effects of mutating the only two non-conserved residues between rat and human ASIC1b in and around the mambalgin binding site: C208Q (equivalent to Arg175 in rASIC1a) and N324K (equivalent to Asn291 in rASIC1a) (Fig. 6a). For rASIC1b C208Q, Ma-3 inhibited pH 5 evoked currents similarly to wild-type rASIC1b, but potentiated pH 6 evoked currents comparable to its effect on hASIC1b, with an alkaline shift in the pH-dependence of activation (Fig. 6d). These effects mostly resemble the effect of Ma-3 on hASIC1b, but we see a greater alkaline shift in the activation curve with the C208Q mutant. Strikingly, this single mutation causes an acidic shift in the activation curve without any peptide present (pH_50 act_: rASIC1b = 6.15, rASIC1b C208Q = 5.84, and hASIC1b = 5.91), further highlighting the importance of this region in activation gating properties. The effect of Ma-3 on rASIC1b N324K was very similar to that on rASIC1b but significantly less potent (Fig. 6e). This is similar to the loss of potency observed in the equivalent mutation when looking at the differences in Ma-3 activity at rat and human ASIC1a (N291K substitution), suggesting that this is also a binding site for Ma-3 in the ASIC1b background. Notably, this loss in potency resulted in Ma-3 inhibiting pH 5 currents with a similar IC_50_ as at hASIC1b. Together this is reflected in the pH-activation data showing no shift in pH_50_ but less inhibition at all activating pH values compared to wild-type rASIC1b, indicating that Asn324 is likely not important in the pH-sensing properties of the channel. Finally, we combined both mutations to make rASIC1b C208Q/N324K (Fig. 6f), resulting in concentration-response curves of Ma-3 with pH 5 and 6 stimuli, as well as a peptide induced shift in the pH-dependence of activation, comparable to wild-type hASIC1b. In summary, these two mutations significantly alter the response of the channel to Ma-3, with the C208Q mutation primarily responsible for causing an alkaline shift in the activation curve, and the N324K mutation reducing the potency of Ma-3. Thus, the unique pharmacology of Ma-3 at hASIC1b is determined by the combined effect of these two substitutions.

### The Ma-3 inhibitory pharmacophore at hASIC1b resembles rASIC1a more than rASIC1b but the potentiating pharmacophore appears to contain less residues

**Fig. 7:**
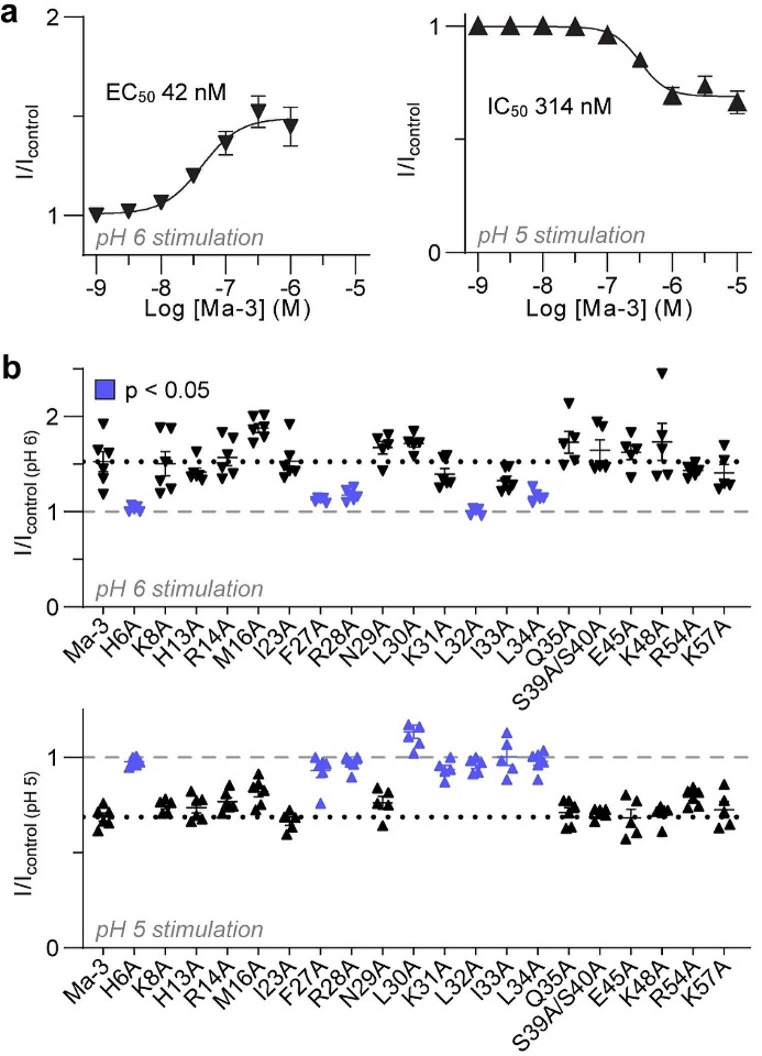
The Ma-3 pharmacophore at hASIC1b. (**a**) Concentration-response curve of Ma-3 at hASIC1b activated by pH 6 and pH 5 (*n* = 5–6). (**b**) Ma-3 and mutants (1 µM) tested for activity at hASIC1b activated by pH 6 (top) and pH 5 (bottom). Violet indicates statistically significant differences from Ma-3 (Welch’s one-way ANOVA with Dunnett’s multiple comparisons test). Data are *n* ≥ 5 with all points shown to give exact replicates. All data use a conditioning pH of 7.45, and are mean ± SEM. See Supplementary Tables 12 and 13 for details of inhibition and statistical comparisons of panel b data

We tested our panel of Ma-3 mutants on both pH 5 and pH 6 evoked currents of hASIC1b using a single concentration of 1 µM, which results in maximal inhibition of pH 5-induced currents and maximal potentiation of pH 6-induced currents (Fig. 7a). Like rASIC1a and rASIC1b, Ma-3 mutants H6A, F27A, R28A, L32A, and L34A lost activity at both pH 5 and pH 6 stimulated currents (Fig. 7b). Interestingly, L30A, K31A, and I33A mutants at 1 µM lost inhibitory activity for pH 5 currents, but still potentiated pH 6 currents like wild-type Ma-3. However, it is important to note that there is a ~ 10-fold difference in potency for wild-type Ma-3 between these pH stimulus conditions, and the activity/potency is highly dependent on the stimulating pH used. Notably, M16A and K8A had no effect on activity for both pH 5 and 6 activity, making this part of the pharmacophore more similar in profile to rat and human ASIC1a than rASIC1b (where these mutations substantially decreased activity; see Fig. 4e). In conclusion, our results suggest that the pharmacophore of Ma-3 at hASIC1b appears to be more like rASIC1a than rASIC1b, despite the higher similarity between ASIC1b species variants than the ASIC1a and ASIC1b subtypes. Interestingly, the more potent effect of potentiating pH 6-induced currents seems to result from a smaller number of interacting residues.

## Discussion

Mambalgins provide potent analgesia in rodents through inhibition of ASIC1a and ASIC1b containing channels [8], and have been used to investigate ASIC1a’s role in cancer cell biology [23–26]. Despite this, the specific molecular determinants of species and subtype dependent activity of these peptides have remained largely unexplored. Previous studies have focused on the interaction of mambalgins with ASIC1a [8, 13–16, 22, 24, 27–34], despite the significant differences in ASIC1b pharmacology [12]. At ~ 6.6 kDa and with a highly charged surface, mambalgins are unlikely to provide analgesia by targeting ASIC1a in the central nervous system if delivered via clinically relevant peripheral administration routes [33]. In contrast, ASIC1b as a peripheral extracellular target presents a more viable option for the development of potential peptide analgesics. Our investigation into the molecular interactions of Ma-3 with ASIC1a and ASIC1b offers important insights into understanding how these allosteric ligands pharmacologically discriminate between closely related channel variants that have distinct biological roles and accessibility.

**Fig. 8:**
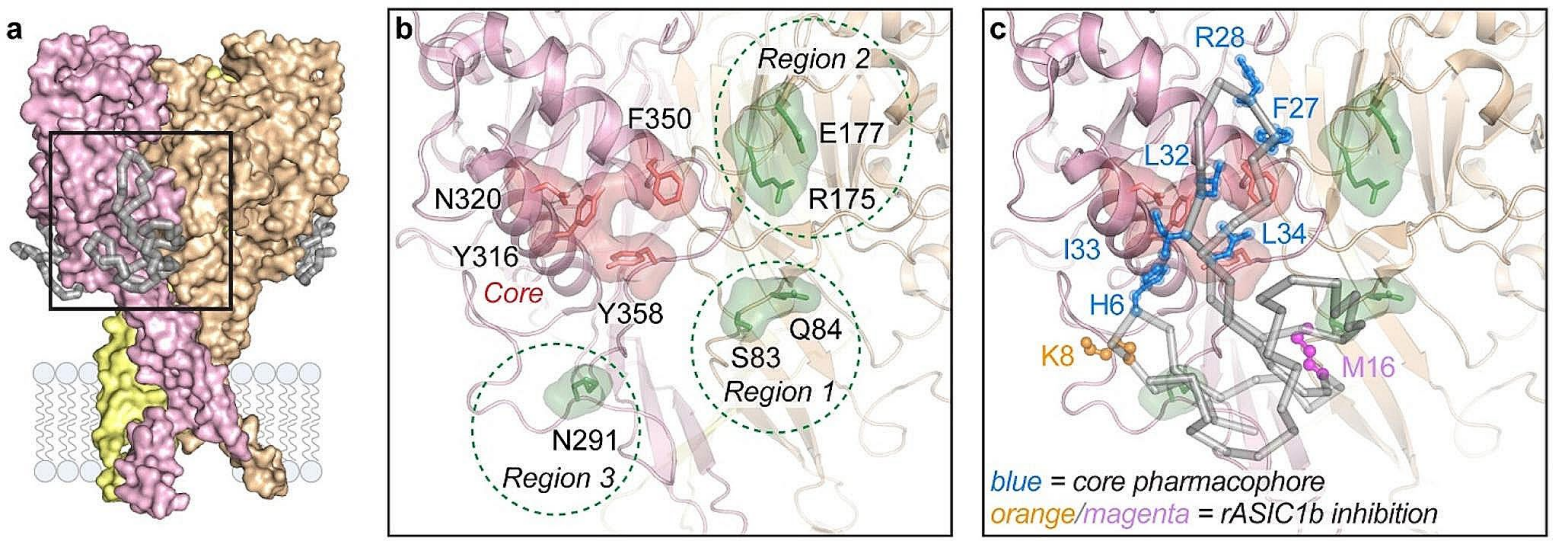
Residues surrounding the core pharmacophore of mambalgins determine selectivity. (**a**) Cryo-EM complex of Ma-1 (grey) bound to hASIC1a trimer (pink, brown, and yellow monomers; PDB: 7CFT). (**b**) Zoomed in inset of black box from panel a, with mambalgin molecules removed. Side chains of the conserved ASIC1 pharmacophore that drives the binding affinity is shown in red. The three peripheral regions identified here that modulate mambalgin activity are shown in green. All side chains are labelled using rASIC1a residues and numbering. (**c**) The same image as in panel b, but also showing Ma-1 (backbone in grey). Mambalgin residues in blue are pharmacophore residues that are important for activity at ASIC1a and ASIC1b. Distant from this cluster are Lys8 (orange) and Met16 (magenta) that are important for the inhibition of rASIC1b but not rASIC1a. See Supplementary Fig. 6 for sequence alignment highlighting positions mutated in this study

Functional and structural studies have previously identified a cluster of tightly packed and conserved residues (Tyr316, Asn320, Phe350, and Tyr358) on the ASIC1 thumb domain as the primary interaction site with mambalgins that confers high binding affinity but does not explain subtype or species dependent pharmacology (Fig. 8) [15, 16, 22]. Here, we identified three spatially separated channel regions that are important determinants of mambalgin activity; region 1, the lower palm containing Ser83 and Gln84; region 2, the upper palm around Arg175 and Glu177; and region 3, the lower thumb with Asn291 (all residues and numbering are rASIC1a; see Supplementary Fig. 6 for rat and human ASIC1 sequence alignment). Region 1 sits ~ 5–10 Å from the β11-β12 linker that includes the highly conserved residues Leu413 and Asn414 in rASIC1a [32, 34–36]. These residues are required for channel desensitisation, with surrounding residues known to alter and fine tune desensitisation kinetics by influencing the β11-β12 linker isomerisation [37]. Conformational changes in the thumb-palm interface, where region 2 is located on the palm, are important for channel activation [37–39]. Engineering of a disulfide bond across the palm: thumb interface (R175C: E355C(E353 in rASIC1a)) led to current inhibition that could be reversed upon application of reducing agents [40]. Furthermore, charge neutralisation and reversal of Arg175 produced acidic shifts in the pH-dependence of activation and steady-state desensitisation (making it more like ASIC1b in terms of pH sensitivity). Glu177 does not seem to play a role in channel gating (a charge neutralisation mutation to Gln had no effect on either activation or SSD of hASIC1a [41], and the effect of substitutions to this site in determining mambalgin selectivity is likely via altering the position of Arg175. This is supported by the observation that the individual mutations and double mutation all had the same effect on Ma-3 function. Our data show that a combination of substitutions across ASIC1 that are involved in gating or repositioning of residues involved in gating, as well as direct binding interactions with mambalgins dictate the observed subtype and species dependent pharmacology.

The effect of Ma-3 on rASIC1a and rASIC1b differs in terms of potency, efficacy, and mechanism of inhibition. Mambalgins stabilise the resting state of ASIC1a by binding to the thumb and preventing the conformational changes associated with activation gating [8, 12]. In contrast, the effect of mambalgins on rASIC1b appears to be more complex, with reports showing a small alkaline shift of variable degree in the SSD curve (comparison of Fig. 2f with previous reports [7] using pH 8 conditioning), and little to no effect on the activation curve. Additionally, Ma-3 alters the rise and decay times of rASIC1b currents but not rASIC1a currents (see Fig. 2). These observations suggest that mambalgins inhibit rASIC1b by disrupting both the activation and desensitisation gating, with the overall outcome further tuned by local pH conditions and receptor occupancy. This model is supported by our data showing that the combined effect of mutating both region 1 (desensitisation) and region 2 (channel activation) are required together to transfer the inhibitory phenotype between rASIC1b and rASIC1a. Interestingly, the significant change in activity and mechanism between rASIC1b and hASIC1b is almost entirely driven by the single region 2 substitution C208Q (equivalent to position 175 in ASIC1a), further highlighting the key role of this channel region and residue in activation gating of ASICs.

We demonstrate that altering intra-ASIC domain interfaces, through substitution mutants and chimeric channels, in positions not directly involved in mambalgin binding can influence peptide pharmacology. These findings are consistent with prior studies using Ma-2 on chimeric ASICs that interchange the thumb, palm and β-ball domains of rASIC1a and rASIC2a. Domain swaps incorporating the regions we identified as important for rASIC1a to rASIC1b pharmacology, led to alterations in mambalgin activity resembling the pharmacologically distinct profiles of Ma-3 across ASIC1 variants. For example, partial block (ASIC1a with ASIC2a palm – covering our region 2) and potentiation of currents (ASIC2a with ASIC1a thumb – combining the ASIC1 primary binding domain with our regions 1 and 2 contributed by ASIC2a) [22]. This suggests that mambalgin binding to the thumb domain can induce distinct conformational changes across different channels depending on how the concerted gating transitions are transduced between domains, which ultimately influences transmembrane pore opening. In examining the effects of the peptide PcTx1 at rASIC1a: ASIC1b chimeras, it was shown that without altering the peptides binding site in the different chimeras, the functional outcome (stabilising the desensitised or open state of channels) depended on channel residues far from the PcTx1 binding site and closer to the transmembrane domain. These findings highlight that mutations and chimeras can induce modifications at distant sites, which can cause significant changes in channel gating and function, in addition to altering the molecular surface presented to ligands for channel binding. While the core binding residues remain intact and potency is largely preserved, these changes may also influence the binding occupancy and/or population of gating states stabilised by mambalgins.

Mambalgin interaction kinetics, which occur over timescales shorter than the standard 45-second application time used here, may lead to incomplete efficacy at saturating concentrations with some channel variants and could be due to the inability to achieve full receptor occupancy under the conditions used. It’s also important to note that each channel has three mambalgin binding sites. Therefore, different receptor-to-bound-peptide ratios could contribute to the varied functional outcomes across subtypes. This complexity is reminiscent of the effects proposed to explain the observed inhibitory and potentiating effects of PcTx1 on ASIC1a [21, 42, 43].

A physiologically relevant example of increased pharmacological complexity due to mixed subunit domain interfaces is seen in heteromeric channels. Mambalgins can modulate ASIC2 and ASIC3 containing heterotrimers provided they include either ASIC1a or ASIC1b [7, 8, 12]. Using standard assay conditions (conditioning pH 7.4 and stimulating pH 5) mambalgins potently inhibit ASIC1a: ASIC2 and ASIC1a: ASIC1b heterotrimers [8]. Interestingly, the effect of mambalgins on ASIC1b: ASIC3 heteromers, as are likely found in sensory neurons, is more complex and exhibits the pH-dependent inhibition and potentiation observed at hASIC1b [7, 12]. Understanding mambalgin binding and pharmacology becomes even more challenging when considering receptor occupancy in these heteromeric channels. This complexity is a crucial factor to consider when using mambalgins in assays where there is variable expression of different ASIC subunits. As such, detailed studies on ASIC heteromers are warranted, especially for any mambalgin analogues deemed suitable for preclinical development.

The core mambalgin pharmacophore is largely shared between rat ASIC1a and ASIC1b. However, the mutation of either Lys8 or Met16 to alanine dramatically alters ASIC1 selectivity. Both mutants showed comparable inhibitory potencies to wild-type Ma-3 against rat and human ASIC1a, but they are largely inactive at rASIC1b. In the cryo-EM model of hASIC1a [15], mambalgin Lys8 is positioned adjacent to a channel region that is identical in sequence between rASIC1a and rASIC1b, thereby unable to readily explain our findings of the K8A mutant. The closest ASIC residue to Met16 is Ser83 of the palm loop in region 1, suggesting they may make interactions in rASIC1b but not in rASIC1a. However, this possibility seems unlikely in light of our data showing that M16A still loses activity at the rASIC1b T128S/E129Q mutant (reversal of rASIC1a S83T/Q84E) mutant. Together, these findings suggest a distinct binding pose at rASIC1b. Our mechanism of action data suggests wild-type mambalgins bind to a different state for rASIC1a (resting) and rASIC1b (desensitised and resting) to produce inhibition. Notably, the chicken ASIC1a structures have been solved in multiple gating conformations. The resting and desensitised states show significant rearrangements in this region, potentially providing a different binding interface to mambalgins and the mutants to interact with. This would require a different mambalgin poses and subtle differences in interacting residues in these lower channel regions. This interpretation is further nuanced by potential structural differences between ASIC1a and ASIC1b. Our current understanding of ASIC structure relies solely on data from chicken and human ASIC1a homotrimers. As such the lack of structural data for ASIC1b represents a crucial gap in our knowledge of ASICs and filling this gap is essential for a comprehensive understanding of ligand selectivity, especially as it pertains to the differential binding of mambalgins to ASIC1a and ASIC1b.

As the K8A and M16A mutants lost little activity at rASIC1a but > 30-fold activity on rASIC1b, they are some of the most rASIC1a selective ligands available and could be useful in future studies as pharmacological tools that better discriminate the contributions of rASIC1a and rASIC1b. For all our mutagenesis data, it is important to consider that substitutions can change the orientation of neighbouring pharmacophore residues, leading mutant mambalgins to interact with channels in a different manner than wild-type combinations. Nevertheless, our Ma-3 mutagenesis results suggest that improvements in ligand selectivity could be obtained by focusing on optimising interactions outside the primary pharmacophore residues. To this end, we have identified Lys8 and Met16 as promising starting points for future extensive structure-activity studies aimed at developing mambalgin analogues with different ASIC1 selectivity profiles and the opportunity to develop ASIC1b inhibitors with less complex mechanism of action.

In conclusion, our analyses demonstrate that a conserved set of pharmacophore residues from ASIC1 and mambalgins play a critical role in providing binding affinity to anchor their interaction, while residues surrounding this core region modulate the relative selectivity profile via allosteric mechanisms. We have shown that single point mutations in both the peptide and channels can elicit diverse, sometimes opposite, changes in pharmacology that ultimately dictate ligand activity. From a therapeutic standpoint, it would be intriguing for future studies to explore whether targeting residues beyond the core binding region of ligands can further enhance specificity across related ion channels. The development of more selective and potent hASIC1b inhibitors will be crucial to improve our understanding of this poorly understood member of the ASIC family. Lastly, our work emphasises the importance of conducting comprehensive mutagenesis studies that encompass species and subtype variants to elucidate the complete pharmacophore. Such investigations offer valuable insights into the intricate molecular interactions underlying ligand selectivity and hold promise for the development of highly selective and potent peptide analogues with potential therapeutic applications.

### Electronic supplementary material

Below is the link to the electronic supplementary material.

Supplementary Material 1

Supplementary Material 2

## Data Availability

The datasets generated during the current study are all depicted/summarised in the main paper and supplementary data file. Raw data sets are available from the corresponding author on reasonable request.
