# Supplementary material for: Revealing molecular determinants governing mambalgin-3 pharmacology at acid-sensing ion channel 1 variants
Source: Cell Mol Life Sci. 2024 Jun 17;81(1):266. doi: 10.1007/s00018-024-05276-2 (PMC11335189; doi:10.1007/s00018-024-05276-2)
Supplement: Supplementary file 2 — Supplementary Material 2 [file 18_2024_5276_MOESM2_ESM.docx]

**Revealing molecular determinants governing mambalgin-3 pharmacology at acid-sensing ion channel 1 variants**

**Ben Cristofori-Armstrong^1,^*, Elena Budusan^2,,4^*, Jennifer J. Smith^3,5^, Steve Reynaud^3,6^, Kerstin Voll^3,7^, Irène R. Chassagnon^3,8^, Thomas Durek^3^, and Lachlan D. Rash^2^**

^1^Australian Institute for Bioengineering and Nanotechnology, ^2^School of Biomedical Sciences, ^3^Institute for Molecular Bioscience, The University of Queensland, St. Lucia, QLD 4072, Australia.

Current Address: ^4^Department of Biomedical Sciences, University of Lausanne, 1011 Lausanne, Switzerland. ^5^Genentech, 1 DNA Way, South San Francisco, California 94080, United States. ^6^In Extenso Innovation Growth, Lyon, France. ^7^Boehringer Ingelheim Pharma GmbH & Co. KG, Birkendorfer Str. 65, 88397 Biberach an der Riß, Germany. ^8^Servatus Ltd. Coolum Beach, QLD, Australia.

*Ben Cristofori-Armstrong and Elena Budusan contributed equally

Correspondence: Lachlan D. Rash; email: [l.rash@uq.edu.au](mailto:l.rash@uq.edu.au), Ben Cristofori-Armstrong; email: b.cristoforiarmstrong@uq.edu.au

## Supplementary information - Figures:


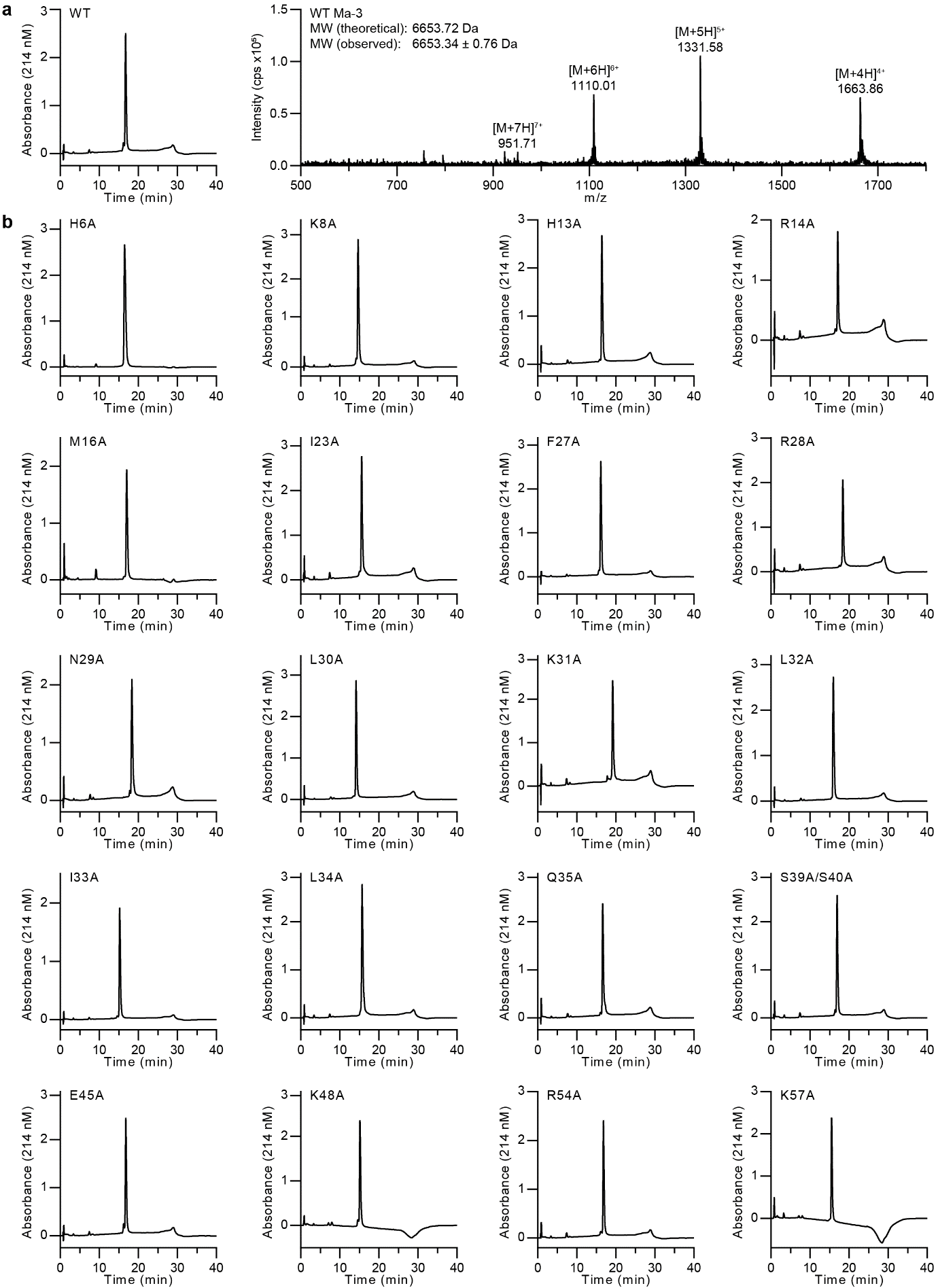


**Supplementary Figure 1. Production of Ma-3 peptides.**

(a) Representative high-performance liquid chromatography (HPLC; left) and electrospray ionisation mass spectrometry (ESI-MS; right) chromatograms of wild-type (WT) Ma-3. (b) HPLC of mutant peptides showing >95% purity.


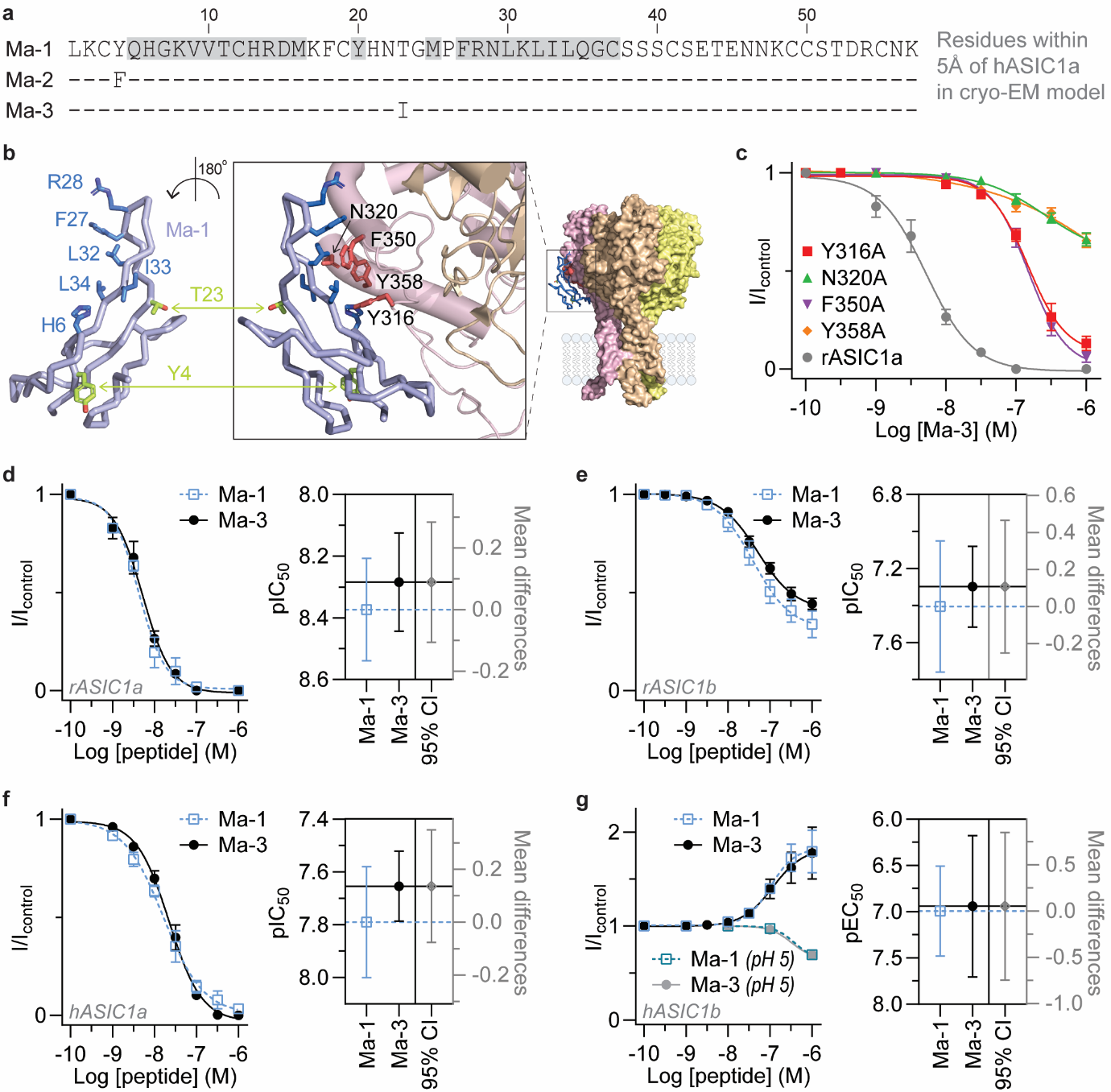


**Supplementary Figure 2. Ma-1 and Ma-3 show no significant difference in ASIC pharmacology.**

(a) Sequence alignment of mambalgins (Ma-1, Ma-2, and Ma-3) with conserved residues as dashes to highlight the Y4F and T23I substitutions from Ma-1. Grey background shading indicates Ma-1 residues within 5Å of hASIC1a in the cryo-EM complex model (PDB 7CFT). (b) The Ma-1 functional pharmacophore at ASIC1a (labelled blue sticks) is spatially clustered. In the complex structure with hASIC1a, these Ma-1 residues make multiple interactions with the core channel residues on the thumb domain that are crucial for inhibitory activity (Tyr316, Asn320, Phe350, and Tyr358 as red sticks and using rASIC1a numbering). Residues that differ between the mambalgins, Y4F and T23I as green sticks, are spatially distant from the peptide pharmacophore, and facing away from ASIC1a in the Ma-1 bound complex. (c) Like Ma-1, concentration-response curves show that Ma-3 loses significant inhibitory activity at core channel pharmacophore mutants. (d–g) Ma-1 and Ma-3 show no significant difference in concentration-response data (P < 0.05) with pH 6 stimulation at (d) rASIC1a, (e) rASIC1b, (f) hASIC1a, and (g) hASIC1b. Statistical comparison via an unpaired two-tailed t-test with Welch’s correction, with full Hill equation fits and statistics reported in Supplementary Table 1. Data are using a conditioning pH of 7.45, and stimulating pH of 6, except for hASIC1b which has additional data with pH 5 stimulation as indicated. All concentration-response data are mean ± SEM and comparison of pIC_50_/pEC_50_ are mean ± 95% confidence intervals (CI), all n = 5–8.


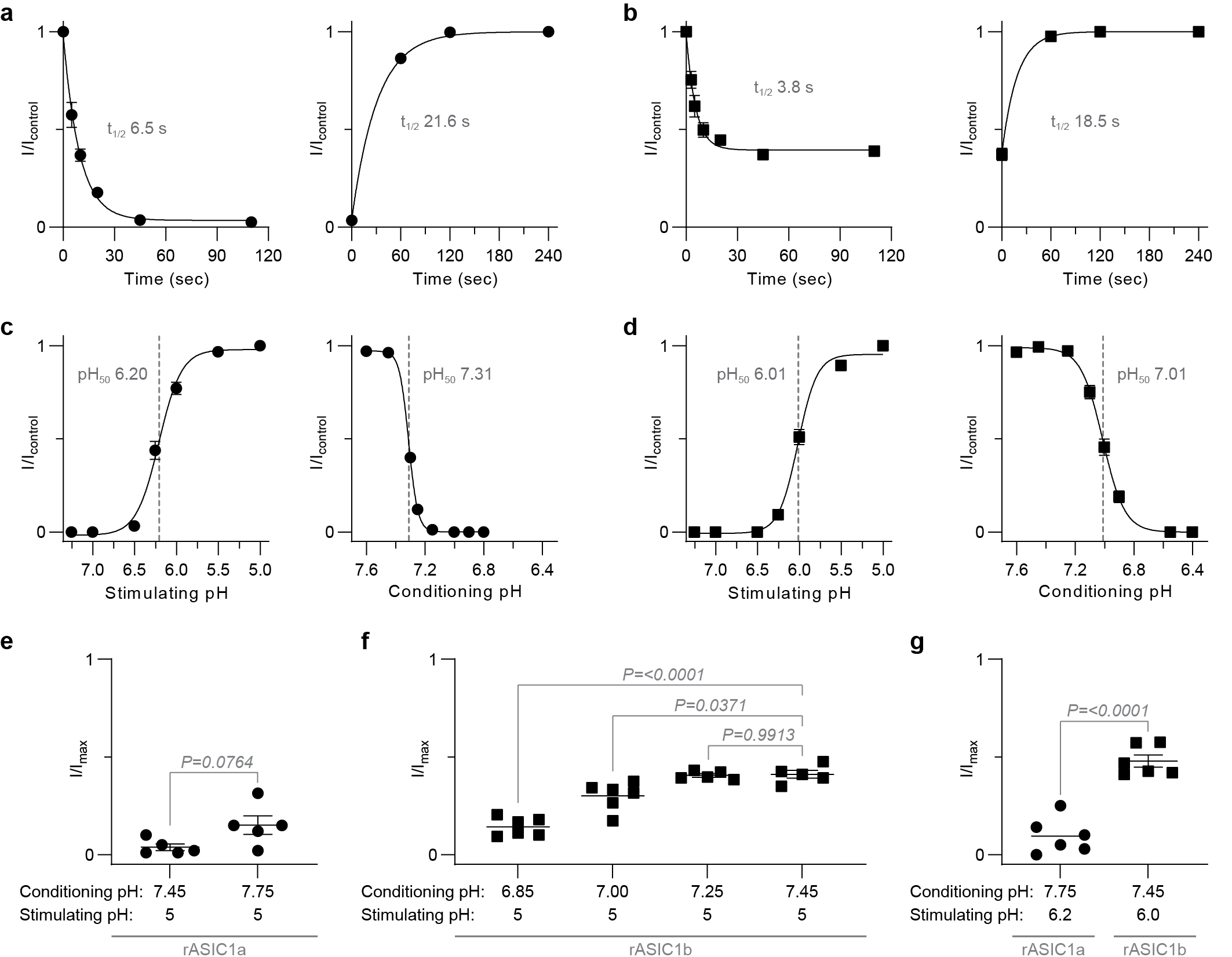


**Supplementary Figure 3. Incomplete inhibition of Ma-3 at rASIC1b is not due to the kinetics of activity or the pH conditions used for testing.**

(a) Left panel shows the peak current amplitude (normalised to current before peptide addition) against the duration of 30 nM Ma-3 application at rASIC1a. Right panel shows the peak current amplitude (normalised to control currents) plotted against time after washing out of Ma-3. (n = 6). Data are fit to a single exponential function to provide the time constants. (b) Plots are as in panel a, however for 300 nM Ma-3 at rASIC1b (n = 5). Panels a and b use a conditioning pH of 7.45 and stimulating pH of 5. (c) rASIC1a and (d) rASIC1b pH-dependence of activation (left; conditioning pH 7.45) and steady-state desensitisation (right; activating pH 5) (n = 3). (e–g) Inhibition by saturating concentrations – 30 nM Ma-3 at rASIC1a (circles) and 300 nM Ma-3 at rASIC1b (squares) – under different conditioning and stimulating pHs as noted (n = 5–6). Pairwise comparisons are done via Welch’s t-test, and statistical tests in panel f via Welch’s one-way ANOVA with Dunnett’s multiple comparisons test where P < 0.05 is considered significant. All data are mean ± SEM.


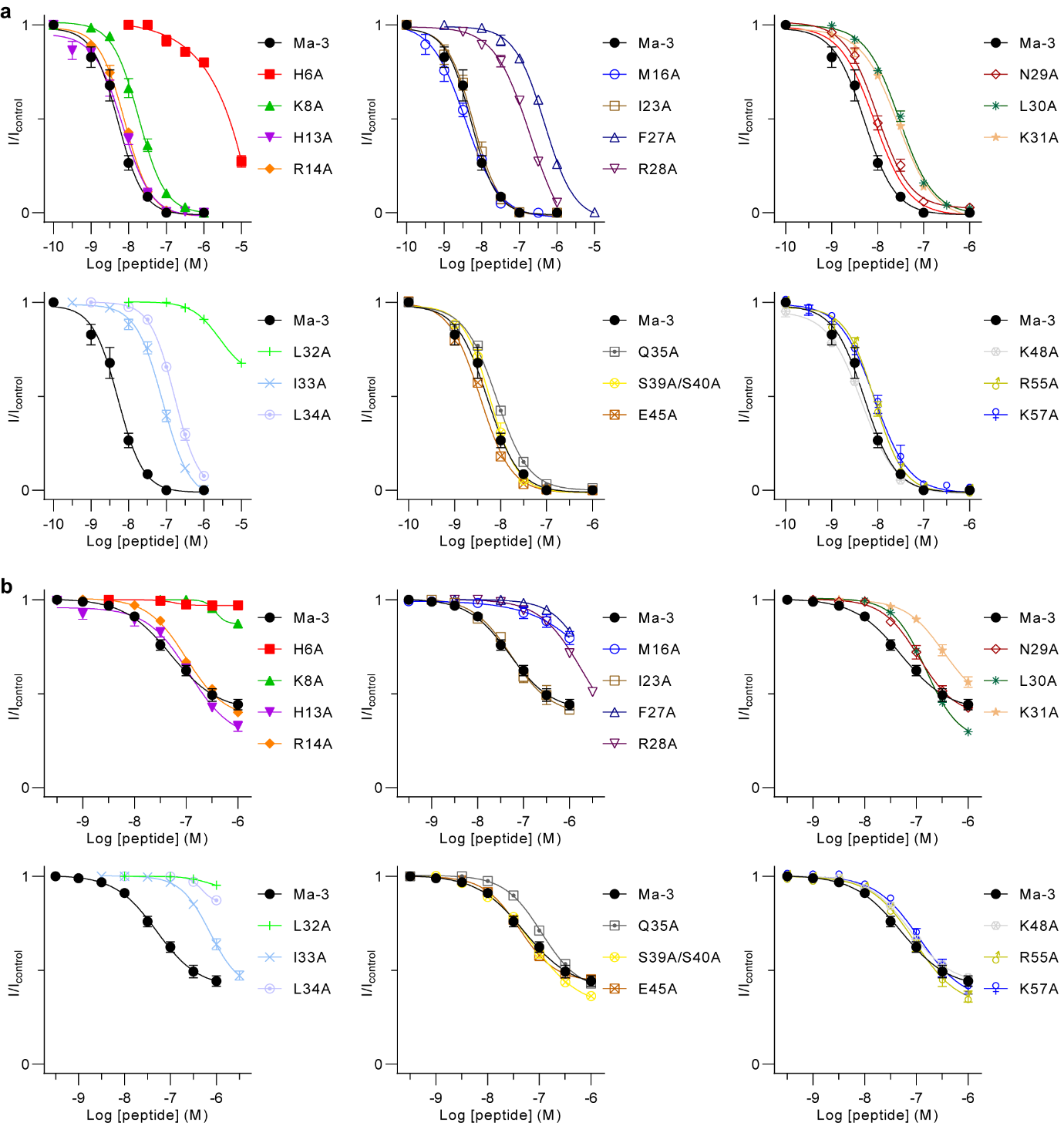


**Supplementary Figure 4. Concentration-response curves of Ma-3 and mutants.**

(a) rASIC1a and (b) rASIC1b data. Data are mean ± SEM and n = 5–6. All data use a conditioning pH of 7.45 and stimulating pH of 6. See Supplementary Table 2 and 3 for full Hill equation fits and statistical comparisons.


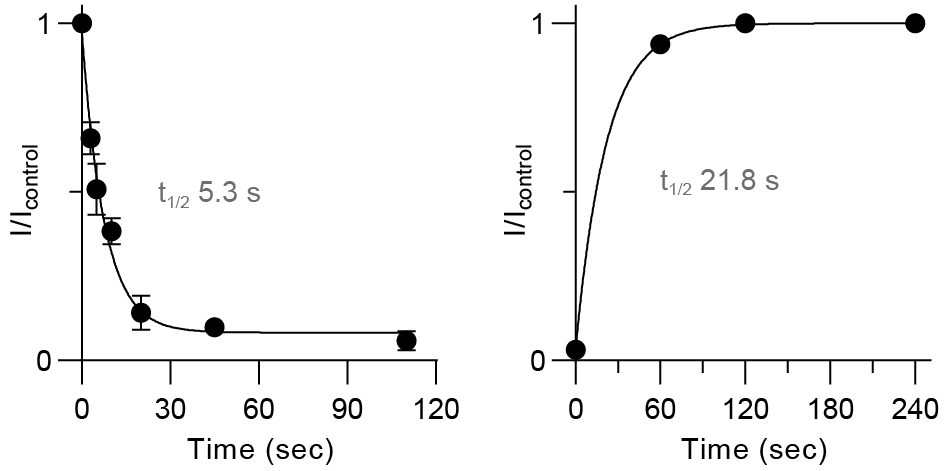


**Supplementary Figure 5. Kinetics of Ma-3 inhibition of hASIC1a.**

Left panel shows the peak current amplitude (normalised to current before peptide addition) against the duration of 100 nM Ma-3 application at hASIC1a. Right panel shows the peak current amplitude (normalised to control currents) plotted against time after washing out of Ma-3. Data are fit to a single exponential function to provide the time constants. Data collected with conditioning pH 7.45 and stimulating pH 5, are mean ± SEM, and n = 5–6.


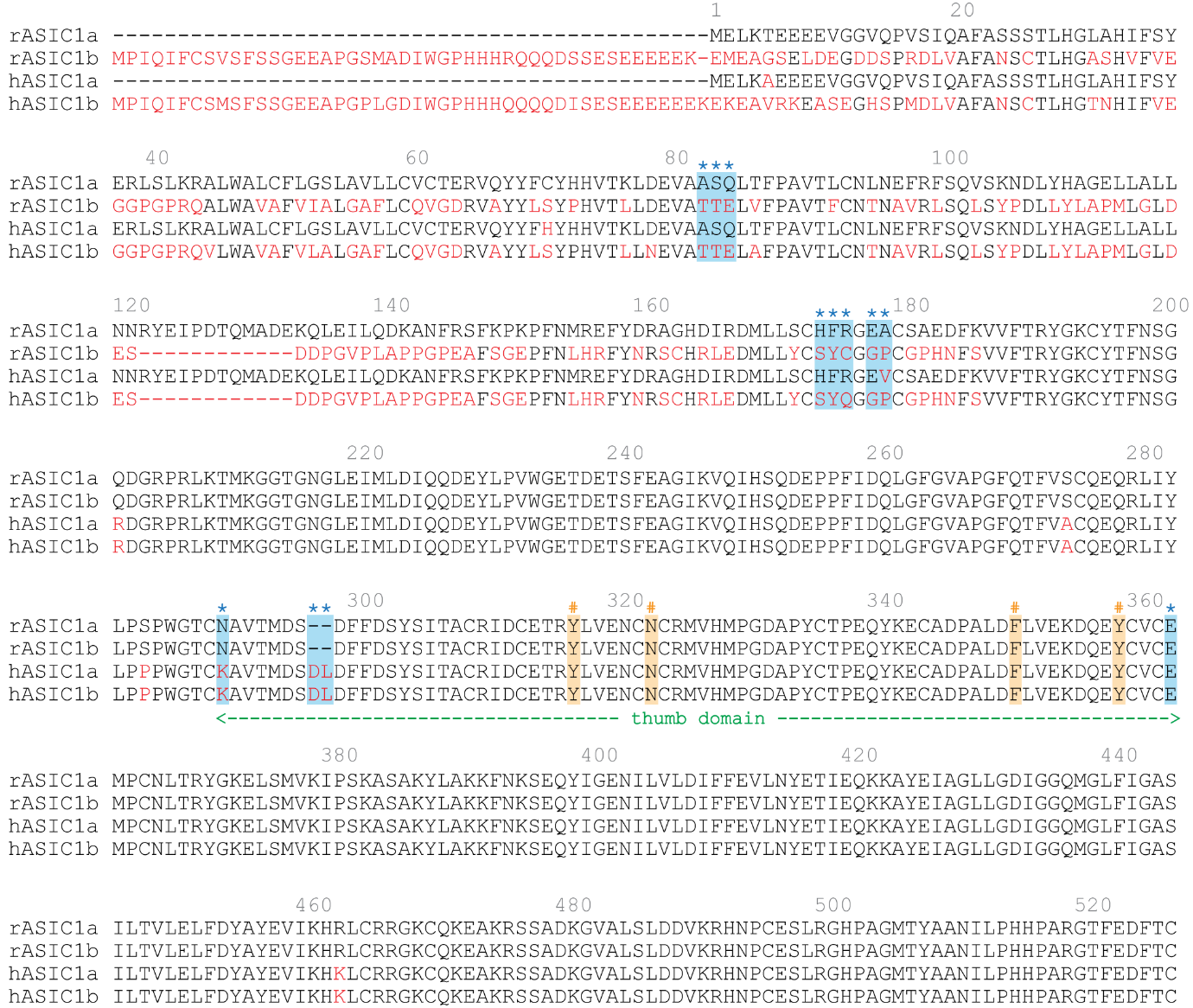


**Supplementary Figure 6. Amino acid sequence alignment of rat and human ASIC1a and ASIC1b.**

Residues that are substituted relative to rASIC1a are shown in red. Blue background shading and a star indicates residues mutated in this study, yellow background shading and an asterisk indicates the four core channel pharmacophore, and the thumb domain is annotated with green text.

**Supplementary information - Tables:**

**Supplementary Table 1. Statistical comparison of Ma-1 and Ma-3 activity at ASIC1 variants.**

Fit of the Hill equation to concentration-response data (pH 6 stimulation) to give IC_50_/EC_50_ and Hill slopes reported as 95% CI. For hASIC1b with pH 5 stimulation, mean and 95% CI of peptide activity also presented. Welch’s t-test compares Ma-1 and Ma-3 pIC_50_/pEC_50_ at different ASIC1 variants; bold values are *P* < 0.05 and considered significant.

| *pH 6 evoked currents* | | | | | |
| --- | --- | --- | --- | --- | --- |
| **channel : peptide** | **IC_50_/*EC_50_**  **(nM)** | **pIC_50_/*pEC_50_**  **(95% CI)** | | ***P* value**  **(to Ma-1)** | **slope**  **(95% CI)** |
| rASIC1a : Ma-1 | 4.24 | 8.50–8.52 | | - | 2.14–0.96 |
| rASIC1a : Ma-3 | 5.19 | 8.16–8.15 | | 0.3310 | 1.87–0.92 |
| rASIC1b : Ma-1 | 39.34 | 7.01–7.64 | | - | 1.64–0.53 |
| rASIC1b : Ma-3 | 50.32 | 7.10–7.45 | | 0.5003 | 1.30–0.70 |
| hASIC1a : Ma-1 | 16.20 | 7.63–7.95 | | - | 1.26–0.66 |
| hASIC1a : Ma-3 | 22.16 | 7.56–7.75 | | 0.1733 | 1.41–0.89 |
| hASIC1b : Ma-1 | *101.66 | *6.73–7.32 | | - | 0.73–2.56 |
| hASIC1b : Ma-3 | *114.90 | *6.51–7.39 | | 0.8814 | 0.59–1.93 |
| *pH 5 evoked currents* | | | | | |
| **channel : peptide** | **I/I_control_** | | **95% CI** | | ***P* value (to Ma-1)** |
| hASIC1b : Ma-1 | 0.67 | | 0.60–0.74 | | - |
| hASIC1b : Ma-3 | 0.69 | | 0.63–0.74 | | 0.6097 |

*indicates current potentiation activity.

**Supplementary Table 2. Effect of Ma-3 on rASIC1a to rASIC1b residue substituted mutants.**

Fit of the Hill equation to concentration-response data with pIC_50_ and Hill slopes reported as 95% CI. Welch’s one-way ANOVA with Dunnett’s multiple comparisons test of pIC_50_ values; bold values are *P* < 0.05 and considered significant.

|  | **IC_50_ (nM)** | **pIC_50_**  **(95% CI)** | ***P* value (rASIC1a)** | ***P* value (rASIC1b)** | **slope**  **(95% CI)** |
| --- | --- | --- | --- | --- | --- |
| rASIC1a | 3.92 | 8.51–8.31 | - | **0.0006** | 1.55–0.94 |
| A82T | 2.63 | 8.80–8.41 | 0.6344 | **0.0015** | 1.38–0.68 |
| S83T | 11.65 | 8.05–7.82 | **0.0016** | 0.1187 | 1.74–0.92 |
| Q84E | 10.20 | 8.07–7.91 | **0.0011** | 0.0792 | 1.41–0.94 |
| H173S | 3.68 | 8.53–8.34 | >0.9999 | **0.0064** | 1.31–0.86 |
| F174Y | 2.48 | 8.74–8.49 | 0.2592 | **0.0017** | 1.19–0.73 |
| R175C | 11.24 | 8.05–7.86 | **0.0006** | 0.1144 | 1.53–0.96 |
| E177G | 18.40 | 7.91–7.56 | **0.0017** | 0.6661 | 1.56–0.69 |
| A178P | 3.68 | 8.55–8.32 | >0.9999 | **0.0072** | 1.22–0.78 |
| RE/CG^1^ | 11.48 | 8.02–7.86 | **0.0006** | **0.0292** | 1.47–0.98 |
| QRE/ECG^2^ | 16.79 | 7.85–7.70 | **0.0002** | 0.1561 | 1.55–1.09 |
| SQRE/TECG^3^ | 20.37 | 7.87–7.53 | **<0.0001** | 0.5530 | 2.08–0.85 |
| rASIC1b | 30.18 | 7.71–7.33 | **0.0006** | - | 1.47–0.68 |

^1^RE/CG is a double mutant combining R175C and E177G in the rASIC1a background.

^2^QRE/ECG is a triple mutant combining Q84E, R175C, and E177G in the rASIC1a background.

^3^SQRE/TECG is a quadruple mutant combining S83T, Q84E, R175C, and E177G in the rASIC1a background

**Supplementary Table 3. Effect of Ma-3 on rASIC1b to rASIC1a residue substituted mutants.** Fit of the Hill equation to concentration-response data with pIC_50_ and Hill slopes reported as 95% CI. Welch’s one-way ANOVA with Dunnett’s multiple comparisons test of pIC_50_ values; bold values are *P* < 0.05 and considered significant.

|  | **IC_50_ (nM)** | **pIC_50_** | ***P* value (rASIC1a)** | ***P* value (rASIC1b)** | **slope**  **(95% CI)** |
| --- | --- | --- | --- | --- | --- |
| rASIC1a | 3.92 | 8.51–8.31 | - | **0.0006** | 1.55–0.94 |
| TE/SQ^1^ | 68.15 | 7.23–7.13 | **<0.0001** | 0.0621 | 1.47–1.22 |
| CG/RE^2^ | 69.34 | 7.26–7.06 | **<0.0001** | **0.0469** | 1.79–1.05 |
| TECG/SQRE^3^ | 18.54 | 7.77–7.70 | **<0.0001** | 0.2504 | 1.29–1.11 |
| rASIC1b | 30.18 | 7.71–7.33 | **0.0006** | - | 1.47–0.68 |

^1^TE/SQ is a double mutant combining T128S and E129Q in the rASIC1b background.

^2^CG/RE is a triple mutant combining C208R and G210E in the rASIC1b background.

^3^TECG/ SQRE is a quadruple mutant combining T128S, E129Q, C208R, and G210E in the rASIC1b background

**Supplementary Table 4. Effect of alanine mutated Ma-3 on inhibition of rASIC1a.**

Fit of the Hill equation to concentration-response data with pIC_50_ and Hill slopes reported as 95% CI. Welch’s one-way ANOVA with Dunnett’s multiple comparisons test of pIC_50_ values; bold values are *P* < 0.05 and considered significant.

|  | **IC_50_ (nM)** | **pIC_50_** | ***P* value** | **slope** |
| --- | --- | --- | --- | --- |
| Ma-3 | 5.19 | 8.42–8.16 | - | 1.87–0.92 |
| H6A | >1000 | - | **<0.0001** | - |
| K8A | 18.53 | 7.80–7.66 | **0.0009** | 1.47–1.03 |
| H13A | 6.90 | 8.26–8.07 | 0.8491 | 1.43–0.90 |
| R14A | 7.92 | 8.16–8.04 | 0.3182 | 1.49–1.09 |
| M16A | 3.71 | 8.57–8.31 | 0.7863 | 1.22–0.75 |
| I23A | 5.92 | 8.34–8.12 | >0.9999 | 1.67–0.96 |
| F27A | 463.57 | 6.39–6.27 | **<0.0001** | 1.27–0.93 |
| R28A | 195.03 | 6.84–6.49 | **<0.0001** | 1.13–0.71 |
| N29A | 9.79 | 8.10–7.92 | 0.0744 | 1.52–0.99 |
| L30A | 29.77 | 7.58–7.48 | **0.0001** | 1.34–1.04 |
| K31A | 27.66 | 7.63–7.49 | **0.0001** | 1.28–0.92 |
| L32A | >1000 | - | **<0.0001** | - |
| I33A | 80.30 | 7.17–7.01 | **<0.0001** | 1.51–0.98 |
| L34A | 166.74 | 6.86–6.67 | **<0.0001** | 1.64–1.06 |
| Q35A | 8.11 | 8.14–8.05 | 0.2489 | 1.40–1.11 |
| S39A/S40A | 5.94 | 8.30–8.16 | 0.9993 | 1.80–1.19 |
| E45A | 3.60 | 8.50–8.39 | 0.4462 | 1.48–1.12 |
| K48A | 4.53 | 8.41–8.28 | 0.9983 | 1.32–1.00 |
| R54A | 8.55 | 8.13–8.01 | 0.1771 | 1.66–1.16 |
| K57A | 8.52 | 8.17–7.97 | 0.2597 | 1.46–0.91 |

**Supplementary Table 5. Effect of alanine mutated Ma-3 on inhibition of rASIC1b.**

Fit of the Hill equation to concentration-response data with pIC_50_ and Hill slopes reported as 95% CI. Welch’s one-way ANOVA with Dunnett’s multiple comparisons test of pIC_50_ values; bold values are *P* < 0.05 and considered significant.

|  | **IC_50_ (nM)** | **pIC_50_** | ***P* value** | **slope** |
| --- | --- | --- | --- | --- |
| Ma-3 | 50.42 | 7.49–7.02 | - | 1.21–0.54 |
| H6A | >1000 | - | **<0.0001** | - |
| K8A | >1000 | - | **<0.0001** | - |
| H13A | 113.69 | 7.10–6.83 | 0.3577 | 1.58–0.84 |
| R14A | 99.74 | 7.14–6.79 | 0.4472 | 1.67–0.78 |
| M16A | >1000 | - | **<0.0001** | - |
| I23A | 54.55 | 7.44–7.03 | >0.9999 | 1.57–0.59 |
| F27A | >1000 | - | **<0.0001** | - |
| R28A | 1840.00 | 6.11–5.71 | **0.0448** | 1.71–0.79 |
| N29A | 110.88 | 7.07–6.79 | 0.2635 | 1.69–0.88 |
| L30A | 151.77 | 6.86–6.80 | 0.0668 | 1.53–1.29 |
| K31A | 337.92 | 6.66–6.15 | **0.0151** | 1.77–0.78 |
| L32A | >1000 | - | **<0.0001** | - |
| I33A | 722.02 | 6.25–5.96 | **0.0002** | 1.77–1.00 |
| L34A | >1000 | - | **<0.0001** | - |
| Q35A | 110.28 | 7.05–6.84 | 0.2258 | 1.51–0.96 |
| S39A/S40A | 68.04 | 7.30–7.03 | 0.9934 | 1.18–0.70 |
| E45A | 41.15 | 7.48–7.29 | 0.9995 | 1.61–0.98 |
| K48A | 73.26 | 7.26–6.98 | 0.9502 | 1.46–0.79 |
| R54A | 91.55 | 7.20–6.88 | 0.6885 | 1.46–0.75 |
| K57A | 113.55 | 7.11–6.74 | 0.3853 | 1.48–0.72 |

**Supplementary Table 6. Effect of Ma-3 wild type, K8A, and M16A mutants on inhibition of hASIC1a.**

Fit of the Hill equation to concentration-response data with pIC_50_ and Hill slopes reported as 95% CI. Welch’s one-way ANOVA with Dunnett’s multiple comparisons test of pIC_50_ values; bold values are *P* < 0.05 and considered significant.

|  | **IC_50_ (nM)** | **pIC_50_** | ***P* value** | **slope** |
| --- | --- | --- | --- | --- |
| Ma-3 | 26.06 | 7.68–7.46 | - | 1.29–0.81 |
| K8A | 93.16 | 7.21–6.71 | **0.0050** | 1.28–0.61 |
| M16A | 27.77 | 7.67–7.43 | 0.9268 | 1.23–0.75 |

**Supplementary Table 7. Effect of 100 nM Ma-3 on the pH-dependence of activation for rASIC1a to hASIC1a.**

Fit of the Hill equation to activation data with pH_50_ and Hill slopes reported as 95% CI. Welch’s t-test of pH_50_ values with and without Ma-3 for each channel; bold values are *P* < 0.05 and considered significant.

|  | **pH_50_** | **pH_50_**  **(95% CI)** | ***P* value**  **(no Ma-3)** | **slope**  **(95% CI)** |
| --- | --- | --- | --- | --- |
| rASIC1a | 6.20 | 6.13–6.26 | - | 2.78–1.47 |
| rASIC1a + Ma-3 | 5.60 | 4.58–5.83 | **0.0121** | 1.84–0.57 |
| hASIC1a | 6.30 | 6.25–6.34 | - | 4.41–2.38 |
| hASIC1a + Ma-3 | 5.86 | 5.65–5.99 | **0.0008** | 1.84–0.86 |

**Supplementary Table 8. Effect of Ma-3 on rASIC1a to hASIC1a residue substituted mutants.**

Fit of the Hill equation to concentration-response data with pIC_50_ and Hill slopes reported as 95% CI. Welch’s one-way ANOVA with Dunnett’s multiple comparisons test of pIC_50_ values; bold values are *P* < 0.05 and considered significant.

|  | **IC_50_ (nM)** | **pIC_50_** | ***P* value (rASIC1a)** | ***P* value (hASIC1a)** | **Slope**  **(95% CI)** |
| --- | --- | --- | --- | --- | --- |
| rASIC1a | 3.92 | 8.51–8.31 | - | **<0.0001** | 1.55–0.94 |
| N291K | 19.11 | 7.79–7.65 | **<0.0001** | 0.2716 | 1.82–1.20 |
| 289-LD | 3.28 | 8.55–8.42 | 0.6861 | **<0.0001** | 1.33–0.98 |
| hASIC1a | 26.06 | 7.68–7.46 | **<0.0001** | - | 1.29–0.81 |
| hASIC1a K291N | 6.34 | 8.33–8.08 | 0.1030 | **<0.0001** | 1.33–0.80 |

**Supplementary Table 9. Effect of Ma-3 on alanine and arginine mutation to rASIC1a E362.**

Fit of the Hill equation to concentration-response data with pIC_50_ and Hill slopes reported as 95% CI. Welch’s one-way ANOVA with Dunnett’s multiple comparisons test of pIC_50_ values; bold values are *P* < 0.05 and considered significant.

|  | **IC_50_ (nM)** | **pIC_50_** | ***P* value** | **slope** |
| --- | --- | --- | --- | --- |
| rASIC1a | 3.82 | 8.52–8.31 | - | 1.53–0.92 |
| E362A | 4.66 | 8.39–8.28 | 0.3897 | 1.51–1.11 |
| E362R | 19.34 | 7.81–7.62 | **<0.0001** | 1.12–0.78 |

**Supplementary Table 10. Effect of Ma-3 for rASIC1b to hASIC1b residue substituted mutants.**

Fit of the Hill equation to concentration-response data with pIC_50_ and Hill slopes reported as 95% CI. Welch’s one-way ANOVA with Dunnett’s multiple comparisons test of pIC_50_ values; bold values are *P* < 0.05 and considered significant.

|  | **IC_50_/**EC_50_* (nM)** | **pIC_50_/*p*EC_50_*** | ***P* value (rASIC1b)** | ***P* value (hASIC1b)** | **slope**  **(95% CI)** |
| --- | --- | --- | --- | --- | --- |
| *pH 5 evoked currents* | | | | | |
| rASIC1b | 4.89 | 7.48–7.10 | - | **0.0126** | 1.59–0.70 |
| C208Q | 59.06 | 7.35–7.08 | 0.9045 | **0.0220** | 1.29–0.77 |
| N324K | 296.50 | 6.73–5.80 | **0.0089** | 0.8800 | 1.86–0.70 |
| CN/QK^1^ | 284.00 | 6.73–5.98 | **0.0044** | 0.8179 | 2.22–0.81 |
| hASIC1b | 313.50 | 6.66–6.35 | **0.0126** | - | 2.98–1.06 |
| *pH 6 evoked currents* | | | | | |
| rASIC1b | 3.39 | 7.71–7.08 | - | 0.9754 | 1.53–0.51 |
| C208Q | **11.13* | **8.34–7.63* | 0.1270 | 0.0668 | 1.26–2.01 |
| N324K | 375.30 | 6.60–5.86 | **0.0010** | **0.0024** | 1.96–0.83 |
| CN/QK^1^ | **31.50* | **7.73–7.27* | 0.9994 | 0.9179 | 0.69–2.48 |
| hASIC1b | **41.90* | **7.66–7.08* | 0.9754 | - | 1.41–1.61 |

^1^CN/QK is a double mutant combining C208Q and N324K in the rASIC1b background.

*indicates current potentiation activity.

**Supplementary Table 11. Effect of Ma-3 on the pH-dependence of activation for rASIC1b to hASIC1b residue substituted mutants.**

Fit of the Hill equation to activation data with pH_50_ and Hill slopes reported as 95% CI. Welch’s t-test of pH_50_ values with and without Ma-3 for each channel; bold values are *P* < 0.05 and considered significant.

|  | **pH_50_** | **pH_50_**  **(95% CI)** | ***P* value (no Ma-3)** | **slope**  **(95% CI)** |
| --- | --- | --- | --- | --- |
| rASIC1b | 6.15 | 6.13–6.16 | - | 5.16–4.09 |
| rASIC1b + Ma-3 | 6.17 | 6.11–6.24 | 0.4549 | 4.90–2.13 |
| C208Q | 5.84 | 5.81–5.86 | - | 2.22–1.86 |
| C208Q + Ma-3 | 6.27 | 6.23–6.31 | **<0.0001** | 3.18–2.12 |
| N324K | 6.19 | 6.16–6.22 | - | 5.18–3.14 |
| N324K + Ma-3 | 6.21 | 6.17–6.25 | 0.5332 | 4.17–2.34 |
| CN/QK^1^ | 5.88 | 5.82–5.93 | - | 2.29–1.59 |
| CN/QK^1^ + Ma-3 | 6.29 | 6.22–6.35 | **<0.0001** | 3.18–1.51 |
| hASIC1b | 5.91 | 5.82–5.99 | - | 3.65–1.67 |
| hASIC1b + Ma-3 | 6.20 | 6.15–6.25 | **<0.0001** | 4.32–2.29 |

**Supplementary Table 12. Effect of alanine mutated Ma-3 on activity of pH 5 evoked hASIC1b currents.**

Mean and 95% CI of peptide activity. Welch’s one-way ANOVA with Dunnett’s multiple comparisons test of I/I_control_ values; bold values are *P* < 0.05 and considered significant.

|  | **I/I_control_** | **95% CI** | ***P* value** |
| --- | --- | --- | --- |
| Ma-3 | 0.69 | 0.63–0.74 | - |
| H6A | 0.98 | 0.95–1.00 | **<0.0001** |
| K8A | 0.75 | 0.70–0.79 | 0.5129 |
| H13A | 0.74 | 0.67–0.80 | 0.9249 |
| R14A | 0.77 | 0.71–0.82 | 0.3027 |
| M16A | 0.80 | 0.73–0.88 | 0.1181 |
| I23A | 0.67 | 0.60–0.73 | 0.9999 |
| F27A | 0.96 | 0.93–1.00 | **<0.0001** |
| R28A | 0.97 | 0.91–1.02 | **<0.0001** |
| N29A | 0.76 | 0.67–0.86 | 0.7117 |
| L30A | 1.16 | 1.07–1.25 | **<0.0001** |
| K31A | 0.94 | 0.90–0.98 | **<0.0001** |
| L32A | 0.96 | 0.91–1.01 | **<0.0001** |
| I33A | 1.00 | 0.88–1.12 | **0.0080** |
| L34A | 0.98 | 0.92–1.04 | **<0.0001** |
| Q35A | 0.71 | 0.64–0.78 | >0.9999 |
| S39A/S40A | 0.71 | 0.68–0.73 | 0.9985 |
| E45A | 0.68 | 0.56–0.81 | >0.9999 |
| K48A | 0.70 | 0.66–0.75 | >0.9999 |
| R54A | 0.79 | 0.75–0.84 | 0.0554 |
| K57A | 0.73 | 0.61–0.84 | 0.9990 |

**Supplementary Table 13. Effect of alanine mutated Ma-3 on activity of pH 6 evoked hASIC1b currents.**

Mean and 95% CI of peptide activity. Welch’s one-way ANOVA with Dunnett’s multiple comparisons test of I/I_control_ values; bold values are *P* < 0.05 and considered significant.

|  | **I/I_control_** | **95% CI** | ***P* value** |
| --- | --- | --- | --- |
| Ma-3 | 1.61 | 1.40–1.82 | - |
| H6A | 1.03 | 0.99–1.07 | **0.0096** |
| K8A | 1.65 | 1.43–1.87 | >0.9999 |
| H13A | 1.42 | 1.30–1.53 | 0.5481 |
| R14A | 1.57 | 1.36–1.78 | >0.9999 |
| M16A | 1.88 | 1.76–2.00 | 0.2390 |
| I23A | 1.59 | 1.39–1.80 | >0.9999 |
| F27A | 1.11 | 1.05–1.16 | **0.0117** |
| R28A | 1.18 | 1.11–1.24 | **0.0252** |
| N29A | 1.71 | 1.62–1.81 | 0.9835 |
| L30A | 1.72 | 1.61–1.84 | 0.9735 |
| K31A | 1.40 | 1.24–1.55 | 0.5381 |
| L32A | 1.00 | 0.96–1.05 | **0.0079** |
| I33A | 1.33 | 1.21–1.45 | 0.1943 |
| L34A | 1.16 | 1.10–1.22 | **0.0209** |
| Q35A | 1.68 | 1.48–1.89 | >0.9999 |
| S39A/S40A | 1.65 | 1.34–1.96 | >0.9999 |
| E45A | 1.63 | 1.41–1.84 | >0.9999 |
| K48A | 1.54 | 1.30–1.77 | >0.9999 |
| R54A | 1.45 | 1.39–1.51 | 0.6585 |
| K57A | 1.47 | 1.25–1.69 | 0.9680 |
